# Oxidative stress-related genes in uveal melanoma: the role of CALM1 in modulating oxidative stress and apoptosis and its prognostic significance

**DOI:** 10.3389/fonc.2025.1618601

**Published:** 2025-08-01

**Authors:** Yue Wu, Xiaoyan Cai, Menghan Hu, Runyan Cao, Yong Wang

**Affiliations:** Department of Ophthalmology, The First Affiliated Hospital of Anhui Medical University, Anhui, China

**Keywords:** uveal melanoma, oxidative stress, CALM1, risk signature, machine learning algorithms

## Abstract

**Background:**

Uveal melanoma (UVM) is a rare yet aggressive form of ocular cancer with a poor prognosis. This study aims to investigate the role of oxidative stress-related genes (OSGs) in UVM, focusing on their involvement in key signaling pathways and immune infiltration and their potential as prognostic biomarkers and therapeutic targets.

**Method:**

Differential gene expression analysis was conducted using 175 samples of normal retinal pigmented epithelium-choroid complex samples and 63 samples from UVM. Protein–protein interaction (PPI) networks were constructed to identify hub genes, and machine learning algorithms were utilized to screen for diagnostic genes, employing methods such as least absolute shrinkage and selection operator (LASSO) regression, random forest, support vector machine (SVM), gradient boosting machine (GBM), neural network algorithm (NNET), and eXtreme gradient boosting (XGBoost). A risk signature model was developed using data from The Cancer Genome Atlas (TCGA) cohort and validated using the International Cancer Genome Consortium (ICGC), GSE84976 dataset. Clinical samples were used to validate the diagnostic value. Experimental validation encompassed H_2_O_2_-induced oxidative stress assays and CALM1 overexpression analysis in UVM cells to evaluate its protective effects.

**Results:**

A total of 2,576 differentially expressed genes (DEGs) were identified, with 185 overlapping OSGs enriched in pathways such as HIF-1, FoxO, PI3K-Akt, and apoptosis. Prognostic hub OSGs, including ACACA, CALM1, and DNM2, were associated with poor survival outcomes in the training set and multiple validation data. Revalidation using clinically collected samples confirmed that CALM1 exhibits superior diagnostic value. The risk signature model demonstrated strong predictive accuracy for a 5-year overall survival (AUC = 0.844). Immune infiltration analysis revealed increased CD4^+^ memory-activated T cells and mast resting cells in the high-risk group. Additionally, CALM1 overexpression attenuated H_2_O_2_-induced oxidative stress and apoptosis in UVM cells. CALM1 upregulation also mitigated the inhibitory effects of H_2_O_2_ on key cellular processes, including proliferation, migration, and invasion.

**Conclusion:**

This study underscores the critical role of OSGs in the progression of UVM and their potential as prognostic biomarkers and therapeutic targets. The identified risk signature model and the protective role of CALM1 offer valuable insights for developing targeted therapies and enhancing patient clinical outcomes in UVM.

## Introduction

Uveal melanoma (UVM) is the most common primary intraocular malignancy in adults, accounting for approximately 85% of all ocular melanomas (1). Despite its rarity, UVM presents significant clinical challenges due to its high metastatic potential and poor prognosis, particularly when metastasis occurs—most commonly in the liver (2–4). Unlike cutaneous melanoma, UVM originates from melanocytes within the uveal tract, which includes the iris, ciliary body, and choroid (5, 6). Although early diagnosis and treatment of primary UVM have improved local disease control, effective systemic therapies for metastatic UVM remain lacking (7, 8), underscoring the need for a deeper understanding of its underlying biology and the development of targeted therapeutic approaches. This review aims to provide an overview of the current knowledge on UVM, with a focus on its molecular mechanisms, diagnostic advancements, and emerging treatment strategies.

Oxidative stress—defined by an imbalance between the production of reactive oxygen species (ROS) and the antioxidant defense system—plays a dual role in tumorigenesis and cancer progression (9, 10). On the one hand, excessive ROS can induce DNA damage, genomic instability, and mutations, contributing to cancer initiation and promotion (11). ROS are also capable of triggering cell death through oxidative stress in various cancers (12). On the other hand, cancer cells often exploit elevated ROS levels to promote proliferation (13), survival (11, 14), and metastasis (15) by activating oncogenic signaling pathways such as PI3K/AKT (14), MAPK (16), and NF-κB (17, 18). Studies have shown that elevated oxidative stress contributes to the anticancer activity of UVM cell lines (19). Recent research has also emphasized the complex interaction between oxidative stress and the tumor microenvironment (TME), wherein ROS modulate immune cell function, angiogenesis, and extracellular matrix remodeling, thereby influencing tumor progression and resistance to therapy. For instance, studies have demonstrated that reactive oxygen species play critical roles in enhancing antigen presentation, regulating immune responses, and preventing immune escape in gastric cancer (20). Targeting oxidative stress pathways—whether by antioxidants or ROS-inducing agents—has thus emerged as a promising therapeutic strategy. However, the context-dependent roles of ROS in cancer demand a nuanced approach to leveraging oxidative stress therapeutically without inadvertently promoting tumorigenesis.

In the present study, we hypothesized that a comprehensive oxidative stress-related gene signature could serve as a prognostic biomarker for patients with UVM. Therefore, differentially expressed genes and a protein–protein interaction (PPI) network were analyzed, followed by machine learning approaches to identify oxidative stress-related prognostic genes. The prognostic efficacy of these genes was then evaluated through a risk signature model. The correlation between risk scores and immune cell infiltration, as well as immune score, was assessed using the Cell-type Identification By Estimating Relative Subsets Of RNA Transcripts (CIBERSORT) algorithm. Additionally, the roles of CALM1 in oxidative stress and apoptosis in UVM cells were validated through cytological experiments.

## Methods

### Data acquisition and performance

RNA sequencing data from 80 UVM patients, along with corresponding clinical information, were collected as the training cohort from The Cancer Genome Atlas (TCGA) database (https://www.cancer.gov/tcga). Dysregulated gene expression was analyzed using datasets from the Gene Expression Omnibus (GEO), including GSE22138 with 63 UVM samples and GSE29801 with 175 normal retinal pigmented epithelium-choroid complex samples. A validation cohort consisting of 370 UVM patients was obtained from the International Cancer Genome Consortium (ICGC) database (https://www.icgc-argo.org). Another validation GSE84976 database was further used to evaluate the prognostic analysis of individual genes, including 28 UVM patients.

Additionally, a total of three UVM tumor samples and matched adjacent normal tissues were collected as clinical validation samples. RNA sequencing (RNA-seq) of tissue samples was performed on an Illumina Nova X Plus (Novogene, Beijing, China) using a paired-end approach with 150-bp reads. The fragments per kilobase of transcript per million mapped reads (FPKM) of genes in clinical samples, including three UVM tumor samples and matched adjacent normal tissues, were provided in **Supplementary Table 1**. All human samples used in this study were obtained with written informed consent from participating patients, in accordance with the ethical guidelines of The First Affiliated Hospital of Anhui Medical University. Specimens were anonymized and handled in compliance with the Declaration of Helsinki.

All data analyses were performed using R software (version 4.1.0) and associated Bioconductor packages. Additionally, 1,065 oxidative stress-related genes (OSGs) were retrieved from previous literature for further investigation (see **Supplementary Table 2**).

### Identification of dysregulated OSGs

A total of 63 UVM samples from the GSE22138 dataset and 175 normal retinal pigmented epithelium-choroid complex samples from GSE29801 (21) were included in the analysis. To minimize batch effects in the combined RNA sequencing data from GSE22138 and GSE29801, the “normalizeBetweenArrays” function from the limma R package and the “ComBat” function from the sva R package were used. Differentially expressed genes (DEGs) between UVM and normal samples were identified using limma, with a significance threshold of *P <*0.01 and |log_2_(fold change)| >1.5. Dysregulated OSGs were visualized in a Venn plot using the VennDiagram R package. Furthermore, Kyoto Encyclopedia of Genes and Genomes (KEGG) pathway enrichment analysis was performed using the KEGG Orthology Based Annotation System (KOBAS) database (http://bioinfo.org/kobas/).

### Network of protein–protein interactions

A PPI network was constructed using the STRING database (http://string-db.org/) (22). Subclusters within the PPI network of OSGs were created to identify candidate hub genes for further analysis, using a median degree threshold (degree cutoff > 61). The subclusters were visualized using the Cytoscape software (version 3.8.2).

### Prognostic risk signature model

Multivariate Cox regression analysis was conducted using the survminer R package to refine the set of OSGs with the best predictive performance. The formula for the risk signature model was defined as follows: risk score = Exp^DNM2^ × 0.133679 + Exp^POMC^ × 0.591935 + Exp^HSP90B1^× + Exp^POMC^ × 0.591935 + Exp^CALM1^ × 0.221127 + Exp^ACACA^ × 0.889051. All UVM patients were classified into high-risk and low-risk groups based on the median risk score (0.960617). Kaplan–Meier survival curves were generated using the survminer package. Additionally, a receiver operating characteristic (ROC) curve was plotted using the survivalROC package to assess the predictive accuracy of the prognostic model.

### Clinical relevance and nomogram development

Next, we investigated the relationship between OSG-related risk signature and clinicopathological characteristics, including age, gender, race, and stage. The “rms” R package was used to develop the nomogram to illustrate each patient’s 1-, 3-, and 5-year overall survival probability, integrating OSG-related risk signature and clinicopathological features. Calibration curves were used to confirm the consistency between the predicted and the actual overall survival.

### Immune cell infiltration

To explore the mechanisms underlying the prognostic impact of OSGs in UVM, immune cell infiltration analysis was conducted using the Cell-type Identification By Estimating Relative Subsets Of RNA Transcripts (CIBERSORT) algorithm. Furthermore, the Estimation of STromal and Immune cells in MAlignant Tumor tissues using Expression data (ESTIMATE) algorithm was employed to compute immune and stromal scores based on gene expression data, using the estimate R package. Correlations between these scores and either individual risk scores or specific hub genes were then assessed.

### Machine learning analysis

Six machine learning algorithms were applied to identify key genes with significant predictive power. The least absolute shrinkage and selection operator (LASSO) method, implemented via the glmnet R package, performed sparse regularization to select crucial genes. Random forest analysis was carried out using the randomForest package to determine gene importance via ensemble learning with decision trees. Support vector machine (SVM) regression, implemented with the e1071 package, contributed to gene selection. Gradient boosting machine (GBM), using the gbm package, applied a sequential ensemble method to screen for predictive genes. Extreme gradient boosting (XGBoost), accessed through the xgboost package, employed advanced tree penalization to refine gene selection. A neural network (NNET) model was constructed using the nnet package. All models were built using 10-fold cross-validation. Model performance on the validation set was evaluated using the mlr3 package, with predictive accuracy quantified by the area under the ROC curve (AUC), calculated using the pROC R package.

### Cell culture and transfection

The UVM cell lines (MP65, MM28) were provided by the Stem Cell Bank, Chinese Academy of Sciences. All cells were cultured in Roswell Park Memorial Institute (RPMI)-1640 medium supplemented with 20% fetal bovine serum (FBS, #C0226, Beyotime Biotechnology, China) and 1% penicillin/streptomycin (#C0222, Beyotime Biotechnology, China) at 37°C in a humidified atmosphere containing 5% CO_2_. UVM cells were treated with hydrogen peroxide (H_2_O_2_) at concentrations of 50, 100, and 200 µmol/L (Merck KGaA, Darmstadt, Germany) in serum-free RPMI-1640 medium for 24 h. The CALM1 overexpression vector (oeCALM1) and a negative control vector were synthesized by GenePharma (Shanghai, China). Transfection was performed using Lipofectamine™ 2000 (Invitrogen, Carlsbad, USA) according to the manufacturer’s instructions. The medium was replaced three times per week, and cells were passaged upon reaching confluence.

### Protein extraction and Western blot

Cells were harvested and washed twice with ice-cold phosphate-buffered saline (PBS) to remove residual medium. They were then lysed in radioimmunoprecipitation assay (RIPA) buffer containing 1% protease inhibitor cocktail. The supernatant containing total protein was collected, and protein concentration was determined using a bicinchoninic acid (BCA) protein assay kit (#P0012, Beyotime Biotechnology, China). Equal amounts of protein (20 µg) were mixed with 4× Laemmli buffer and loaded onto a 10% SDS-polyacrylamide gel (SDS-PAGE). After electrophoresis, proteins were transferred to a polyvinylidene difluoride (PVDF) membrane using a wet transfer system. The membrane was blocked in 5% non-fat dry milk diluted in Tris-buffered saline with Tween-20 (TBST; 20 mM of Tris, 150 mM of NaCl, 0.1% Tween-20, pH 7.6) for 1 h at room temperature. The membrane was then incubated overnight at 4°C with primary antibodies diluted in blocking buffer at the following concentrations: 1:1,000 for anti-GAPDH, 1:2,000 for anti-SOD2, 1:2,000 for anti-CAT, 1:3,000 for anti-CASP3, 1:3,000 for anti-BAX, and 1:2,000 for anti-CALM1. Afterward, the membrane was washed three times for 10 min each with TBST to remove unbound primary antibodies. It was then incubated with horseradish peroxidase (HRP)-conjugated secondary antibodies (anti-rabbit IgG, #A0208, Beyotime Biotechnology, China) diluted 1:200 in blocking buffer for 1 h at room temperature. The membrane was washed three times again with TBST, and protein bands were visualized using an enhanced chemiluminescence (ECL) substrate (#P0018S, Beyotime Biotechnology, China) and detected with a GeneGnome XRQ chemiluminescence imaging system.

### Cell viability assay and ELISA

Cell viability was assessed using the CCK-8 assay (#C0038, Beyotime Biotechnology, China). All UVM cells (MP65 or MM28) were seeded at a density of 5 × 10^4^ cells per well in 96-well plates and transfected with oeCALM1 or the control vector for 24 h. After transfection, 10 µL of CCK-8 reagent was added to each well, and the plates were incubated for 2 h at 37°C in 5% CO_2_. The optical density (OD) at 450 nm was measured using a microplate reader (Thermo Fisher Scientific, USA).

Additionally, the concentrations of superoxide dismutase (SOD, #S0101S), malondialdehyde (MDA, #S0131S), and lactate dehydrogenase (LDH, #P0393S) were measured using ELISA kits (all from Beyotime Biotechnology, China), following the manufacturer’s protocols.

### Transwell for migration and invasion

For migration, we obtained harvest cells (e.g., MP65 or MM28) and resuspended them in serum-free medium and seeded 2 × 10^5^ cells in the upper chamber. The complete medium (with 10% FBS) was added to the lower chamber as a chemoattractant. For oxidative stress conditions, cells were treated with 200 μmol/L of H_2_O_2_ in the upper chamber. For CALM1 overexpression groups, the overexpressing vector and the control negative vector were transfected into cells prior to seeding. Then, all cells were incubated for 48 h at 37°C with 5% CO_2_. Next, we removed non-migrated cells from the upper chamber with a cotton swab. The migrated cells were fixed with 4% paraformaldehyde and stained with 0.1% crystal violet. The images were captured under a microscope, and cell counts were determined by analyzing three random fields per insert. For the cell invasion assay, the same steps as above were followed, but the transwell membrane was precoated with Matrigel (50 μg/mL, (BD Biosciences, San Jose, USA)) to simulate extracellular matrix barriers. All the cells were solidified by Matrigel for 4 h at 37°C before cell seeding. After incubation for 48 h, a microscope was used to capture the image.

### Apoptosis assays

Apoptosis was assessed using Annexin V-fluorescein isothiocyanate (FITC)/propidium iodide (PI) flow cytometry, as described in previous studies (23). Cells were stained with an Annexin V-FITC/PI Apoptosis Detection Kit (MedChemExpress, USA, #HY-K1073), and apoptosis was analyzed using a flow cytometer.

### Statistical methods

**Figure 1:**
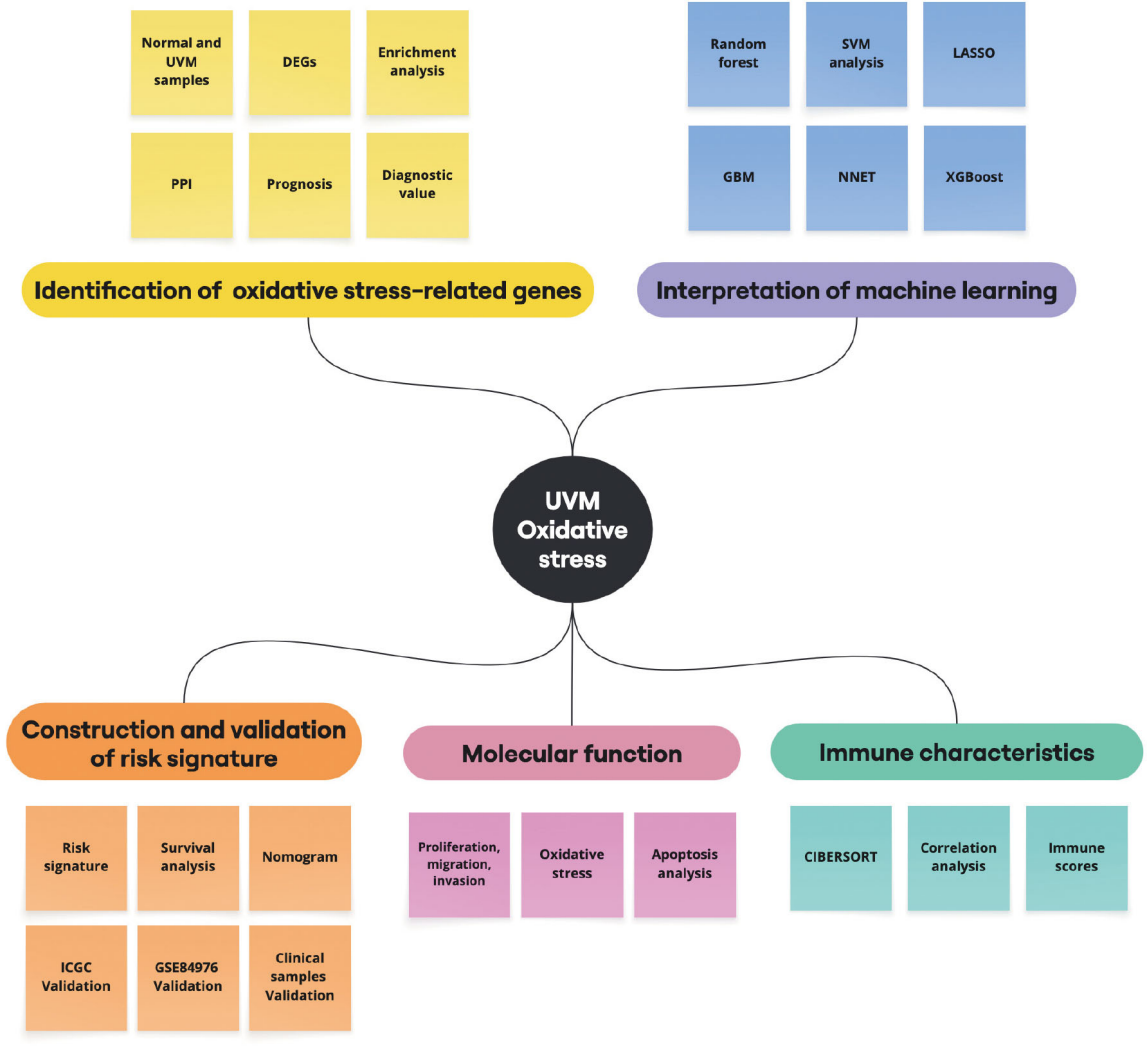
The flowchart of this study.

All results were expressed as mean ± standard deviation (SD). Statistical analyses were conducted using R software (version 4.1.0). Continuous variables were compared using the Wilcoxon rank-sum test. One-way analysis of variance (ANOVA) was applied to determine statistical significance among experimental groups. A *P*-value <0.05 was considered statistically significant unless otherwise indicated. A flowchart of the study design is presented in **Figure 1**.

## Results

### The associated DEGs are implicated in UVM

**Figure 2:**
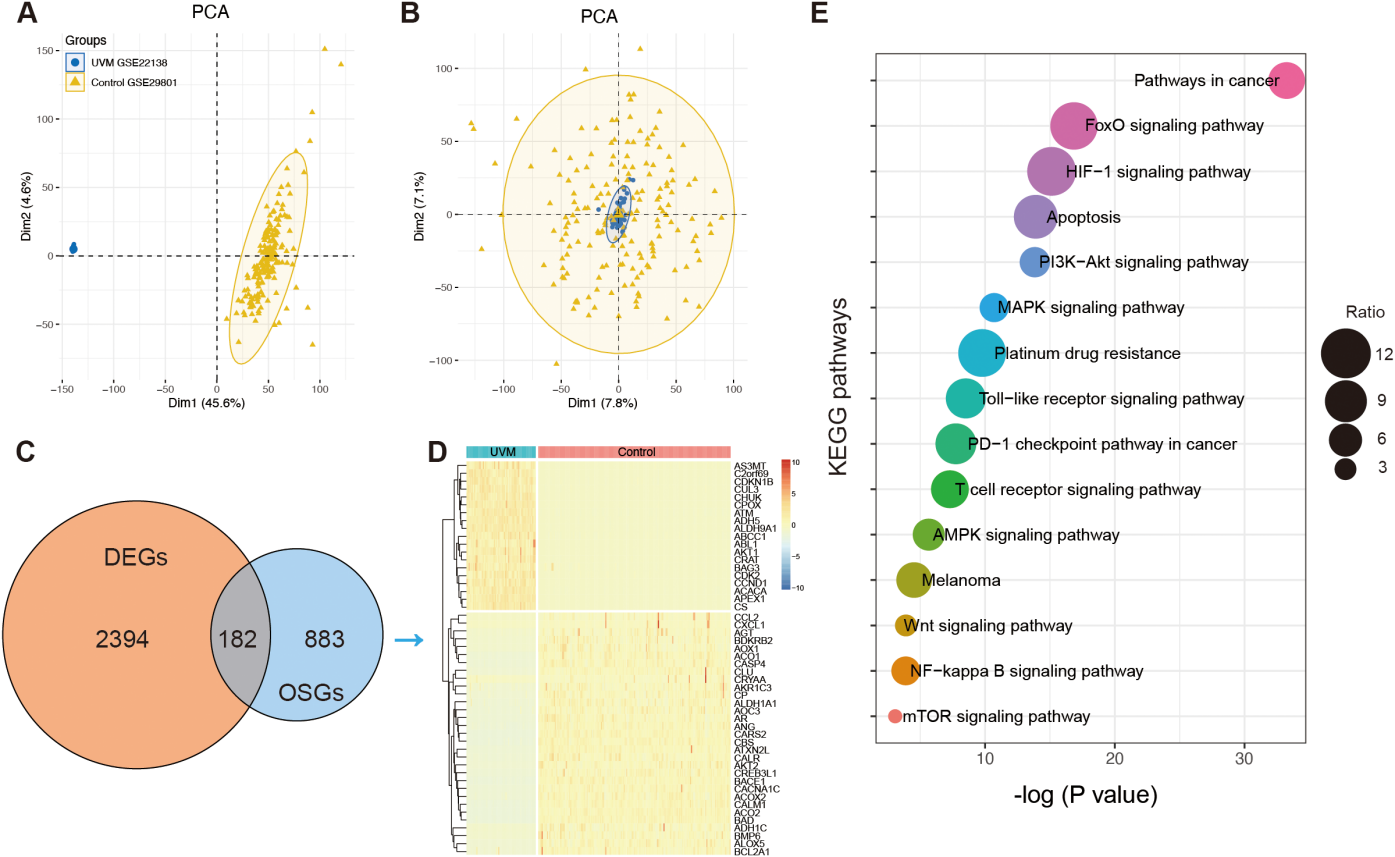
The identification of oxidative stress-related genes (OSGs). **(A)** Principal component analysis (PCA) before batch effects in merging the RNA sequencing data of GSE22138 and GSE29801. **(B)** PCA analysis after batch effects. **(C)** The Venn diagram showing the intersection of differentially expressed genes (DEGs) and OSGs. **(D)** Heatmap showing the differential expression of the intersection of OSGs between normal and UVM samples. **(E)** The enrichment function of Kyoto Encyclopedia of Genes and Genomes (KEGG) analysis.

To analyze the differential expression of OSGs in UVM, 175 normal retinal pigmented epithelium-choroid complex samples from the GSE29801 dataset and 63 UVM samples from the GSE22138 dataset were included in the present study after removing batch effects. Principal component analysis (PCA) was conducted to assess batch effect correction (**Figures 2A, B**). A total of 2,576 DEGs were identified, consisting of 1,164 upregulated and 1,412 downregulated genes, using a threshold of *P <*0.01 and |log_2_(fold change)| >1.5 (**Supplementary Table 3**). A Venn diagram showed that 185 genes overlapped between the DEGs and OSGs (**Figure 2C**). These intersection genes were subsequently visualized in a hierarchical clustering heatmap (**Figure 2D**). Enrichment analysis revealed that these dysregulated OSGs were significantly enriched in pathways related to melanoma, cancer, HIF-1 signaling, FoxO signaling, PI3K-Akt signaling, T-cell receptor signaling, PD-L1 expression and the PD-1 checkpoint pathway, and apoptosis (**Figure 2E**).

### Identification of prognostic hub OSGs in UVM

**Figure 3:**
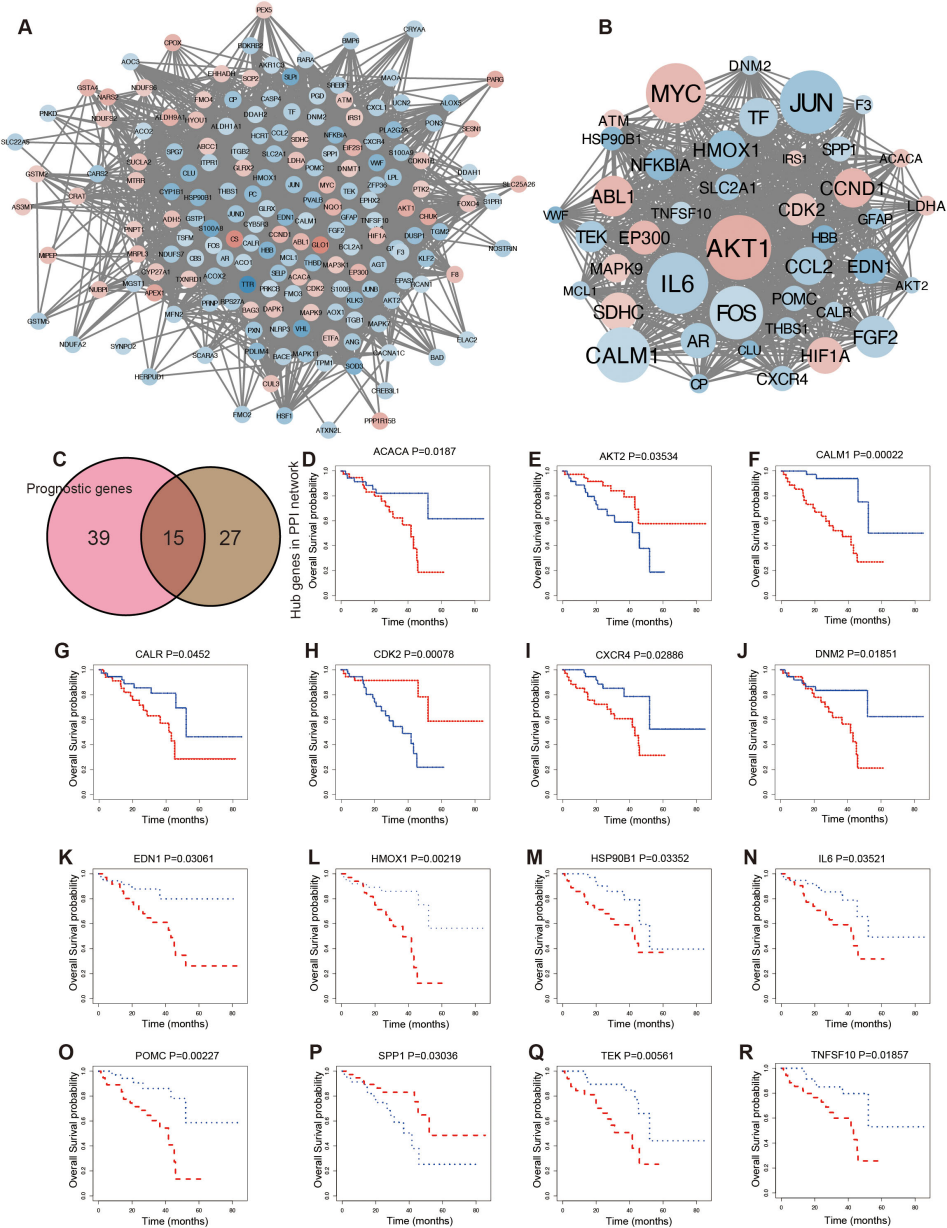
Screening prognostic hub OSGs in UVM. **(A)** The protein–protein interaction (PPI) analysis. **(B)** The hub genes network. **(C)** The Venn diagram showing the overlapping of prognosis and hub genes of the PPI network. Kaplan–Meier survival curve for overall survival according to the expression levels of OSGs, including ACACA **(D)**, AKT2 **(E)**, CALM1 **(F)**, CALR **(G)**, CDK2 **(H)**, CXCR4 **(I)**, DNM2 **(J)**, EDN1 **(K)**, HMOX1 **(L)**, HSP90B1 **(M)**, IL6 **(N)**, POMC **(O)**, SPP1 **(P)**, TEK **(Q)**, and TNFSF10 **(R)**.

To further investigate the interactions among OSGs, the dysregulated OSGs identified above were analyzed using the STRING database to construct a PPI network. The resulting network revealed extensive interactions among dysregulated OSGs (**Figure 3A**). To identify hub genes, subclusters of the PPI network were generated using a median degree cutoff (>61), resulting in 42 hub OSGs for subsequent analysis (**Figure 3B**). In addition, 54 prognostic genes were identified based on Kaplan–Meier survival curves. The intersection of hub genes from the PPI network and prognostic genes yielded 15 common genes (**Figure 3C**). Kaplan–Meier survival analysis demonstrated significant associations between gene expression levels and patient prognosis (**Figures 3D–R**). High expression was strongly correlated with poor survival, including ACACA (**Figure 3D**), CALM1 (**Figure 3F**), CALR (**Figure 3G**), CXCR4 (**Figure 3I**), DNM2 (**Figure 3J**), EDN1 (**Figure 3K**), HMOX1 (**Figure 3L**), HSP90B1 (**Figure 3M**), IL6 (**Figure 3N**), POMC (**Figure 3O**), TEK (**Figure 3Q**), and TNFSF10 (**Figure 3R**). Conversely, low expression of AKT2 (**Figure 3E**), CDK2 (**Figure 3H**), and SPP1 (**Figure 3P**) was associated with poor prognosis.

**Figure 4:**
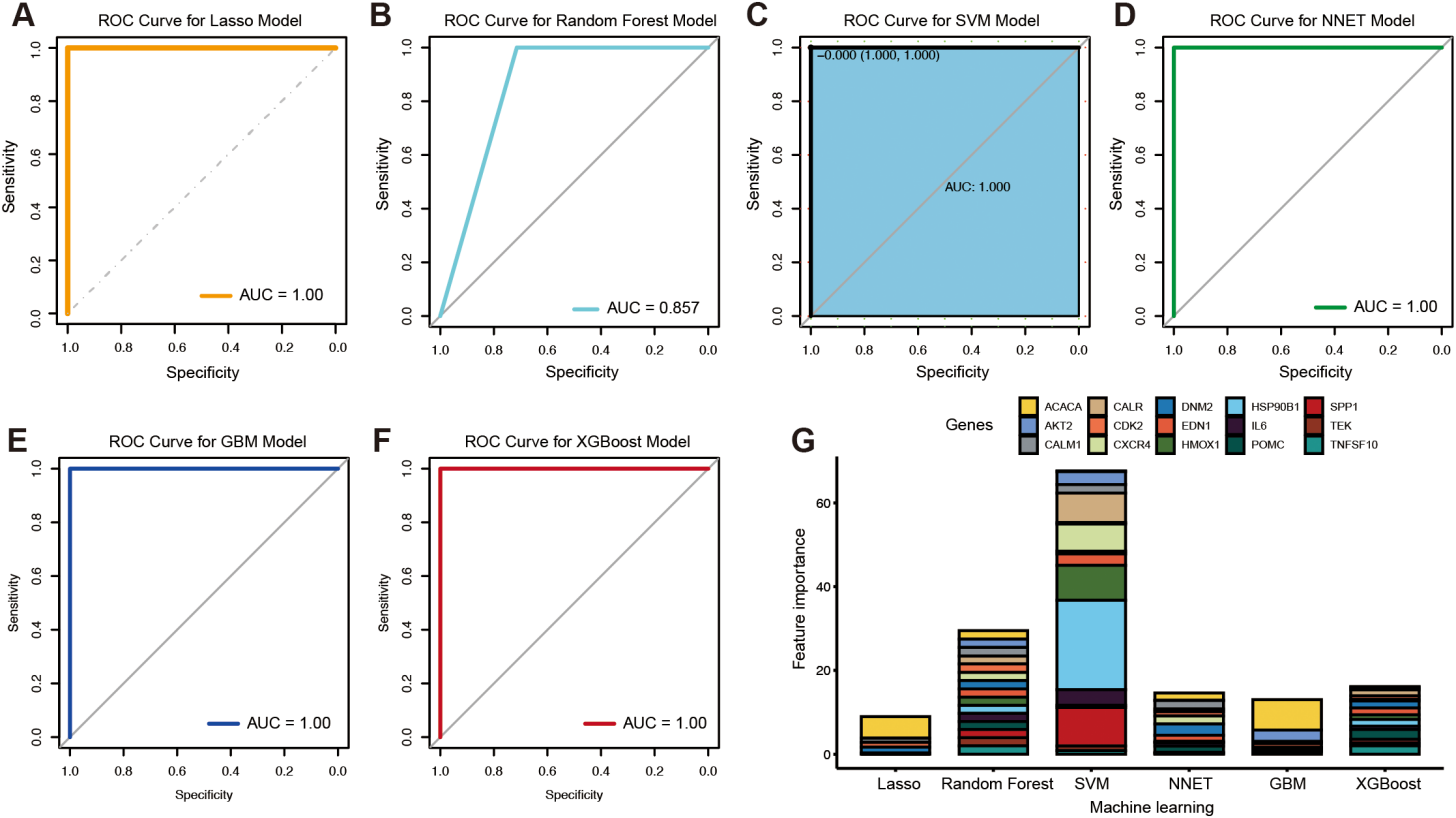
Candidate biomarkers identified by the machine learning algorithms. The ROC curves of the LASSO model **(A)**, random forest **(B)**, SVM **(C)**, NNET **(D)**, GBM **(E)**, and XGBoost **(F)**. The feature importance in different machine learning algorithms **(G)**.

To identify novel genes with diagnostic potential, six machine learning algorithms were applied: LASSO regression, random forest, SVM, NNET, GBM, and XGBoost. ROC curve analysis showed that all models achieved area under the curve (AUC) values exceeding 0.85 (**Figures 4A–F**). Compared with random forest (**Figure 4B**), higher diagnostic potential was observed in the models including LASSO (**Figure 4A**), SVM (**Figure 4C**), NNET (**Figure 4D**), GBM (**Figure 4E**), and XGBoost (**Figure 4F**). Based on feature importance rankings, the top gene identified by each algorithm was selected for further analysis: ACACA (LASSO), CALM1 (random forest), HSP90B1 (SVM), DNM2 (NNET), ACACA (GBM), and POMC (XGBoost) (**Figure 4G**).

### Construction and verification of the OSG-related risk signature

**Figure 5:**
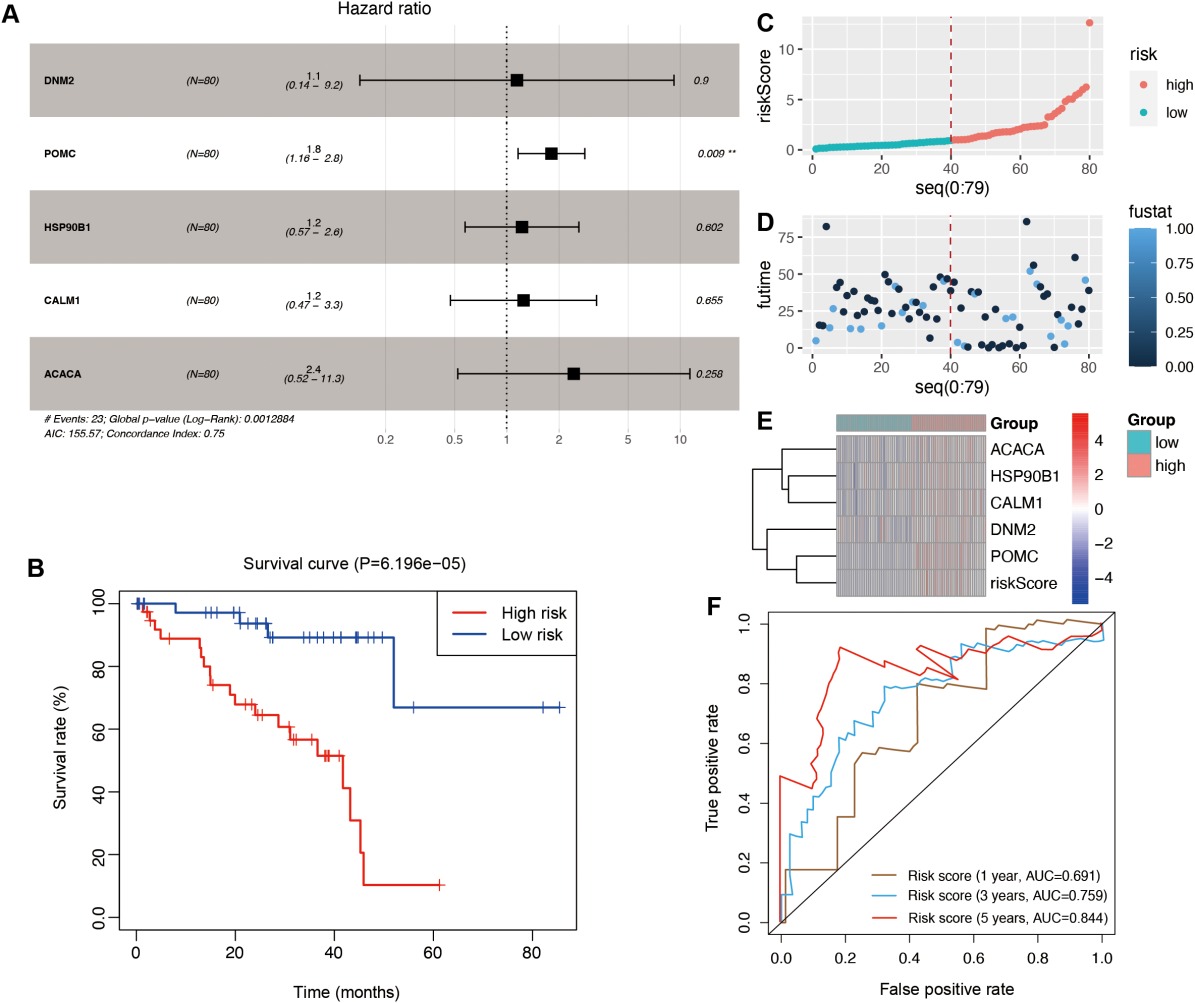
The construction of the risk signature model in the TCGA cohort. **(A)** The multivariate regression analysis. **(B)** Survival curves to evaluate the risk stratification ability of OSGs. **(C)** Risk plots to illustrate the risk scores of different risk groups. **(D)** Risk plots to illustrate the survival status of different risk groups. **(E)** Heatmap showing the expression levels and risk scores in the risk model. **(F)** ROC curves to evaluate the sensitivity and specificity of the risk signature to predict the 1-, 3-, and 5-year overall survival.

**Figure 6:**
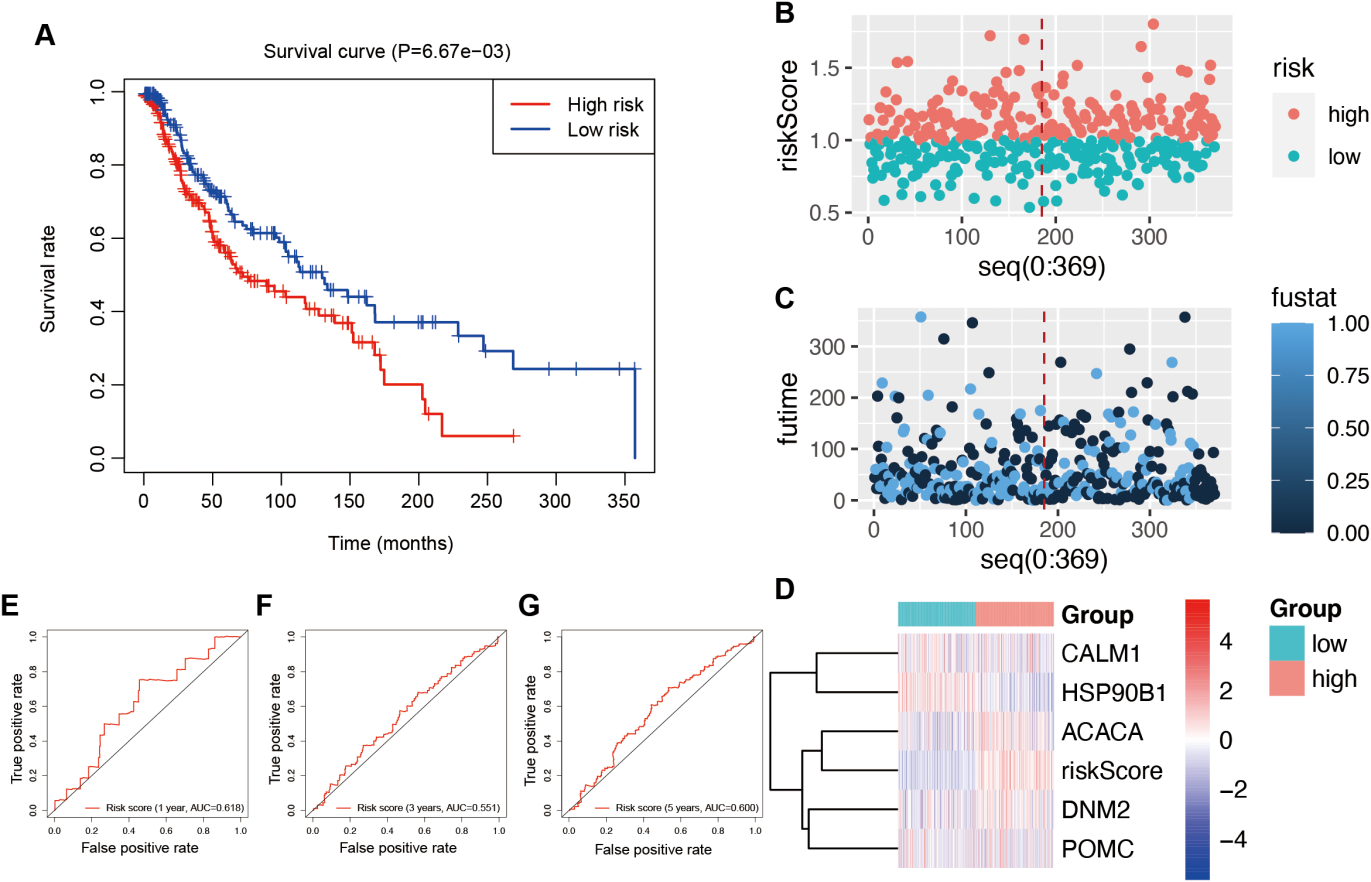
Validation of the risk signature model in the ICGC database. **(A)** Survival curves to investigate the risk stratification ability of OSGs. **(B)** The risk scores of the high- and low-risk groups. **(C)** The survival status of the high- and low-risk groups. **(D)** The expression levels and risk scores in the risk model. ROC curves to evaluate the sensitivity and specificity of the risk signature to predict the 1-year **(E)**, 3-year **(F)**, and 5-year **(G)** overall survival.

Using multivariate Cox regression analysis, a risk signature model was constructed based on the coefficients and expression levels of key genes identified through machine learning (**Figure 5A**). Patients were stratified into high- and low-risk groups according to the median risk score (0.960617). The high-risk group exhibited significantly poorer overall survival compared to the low-risk group (**Figure 5B**), suggesting that the risk score may serve as an independent prognostic factor in UVM. Risk score distribution (**Figure 5C**), survival status (**Figure 5D**), and gene expression profiles (**Figure 5E**) for each patient in the TCGA cohort were also visualized. ROC analysis demonstrated that the model had the highest sensitivity in predicting 5-year overall survival (AUC = 0.844), followed by 3-year (AUC = 0.759) and 1-year (AUC = 0.691) predictions (**Figure 5F**). To validate the model, the ICGC dataset was used in this study. Similar to the training set, the high-risk group in the validation cohort also exhibited significantly worse prognosis (*P* = 6.67e−03; **Figure 6A**). Distributions of risk scores, survival outcomes, and gene expression profiles are shown in **Figures 6B–D**. ROC curves indicated moderate predictive sensitivity for 1-, 3-, and 5-year overall survival (**Figures 6E–G**).

### Association between risk score and immune landscape

**Figure 7:**
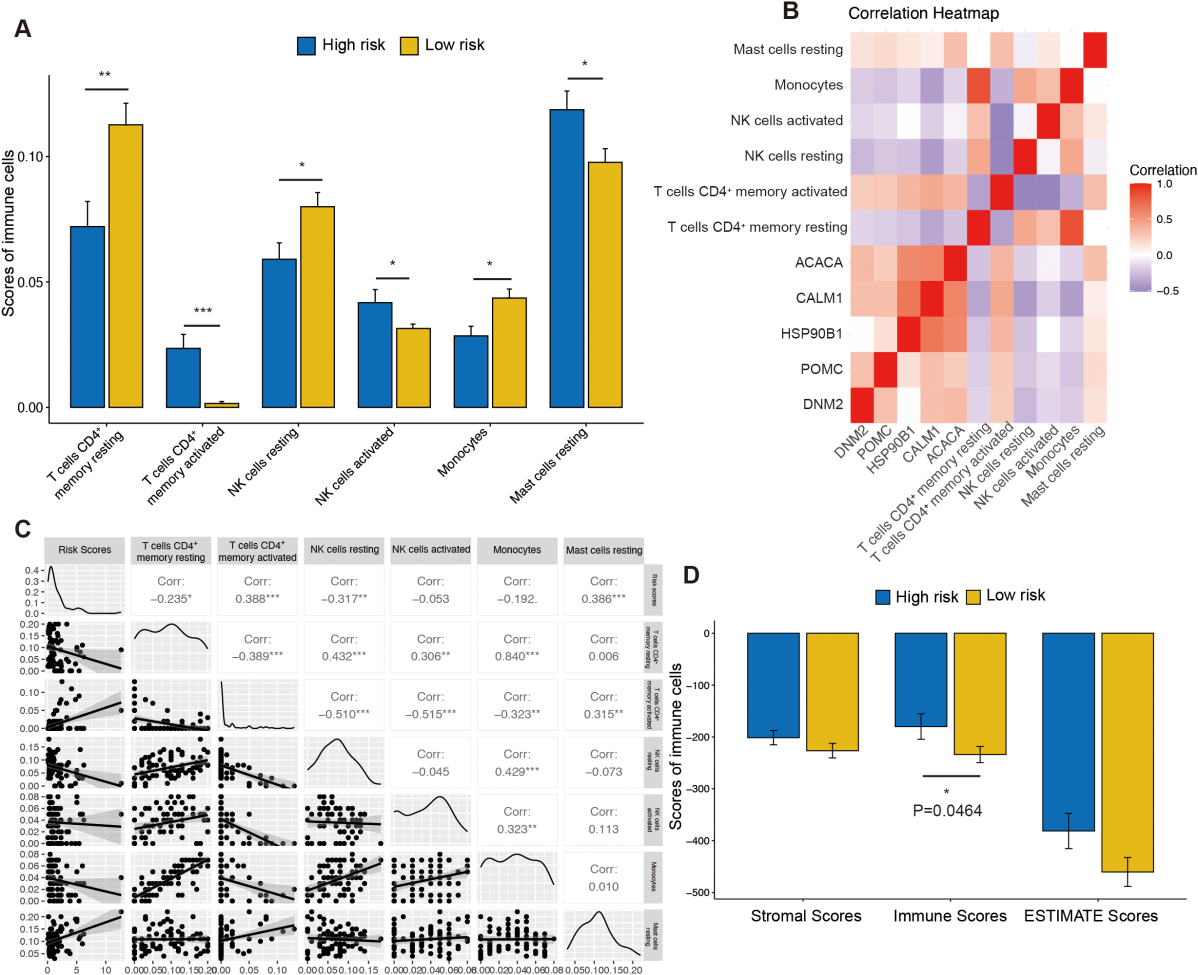
The immune function between the high- and low-risk groups. **(A)** The difference of immune cell infiltration scores between the high- and low-risk groups. **(B)** The heatmap was generated to show the relationship between OSGs and immune cells. **(C)** The correlation analysis between risk scores and immune cells. **(D)** The difference of stromal scores, immune scores, and estimate scores between the high- and low-risk groups. “ns” represents no statistical significance; * represents *P* < 0.05, ** represents *P* < 0.01, and *** represents *P* < 0.001.

To investigate the association between risk score and immune infiltration, the CIBERSORT algorithm was applied. The analysis of immune-related functions revealed that the OSG high-risk group exhibited elevated infiltration scores for CD4^+^ memory-activated T cells, activated NK cells, and resting mast cells (**Figure 7A**). In contrast, the OSG low-risk group showed higher infiltration scores for CD4^+^ memory resting T cells, resting NK cells, and monocytes (**Figure 7A**). Additionally, the correlations between novel OSGs in the risk signature model and immune cell infiltration scores were assessed. The results showed that these OSGs were positively correlated with CD4^+^ memory-activated T cells and resting mast cells (**Figure 7B**). Further analysis revealed that risk scores were positively correlated with CD4^+^ memory-activated T cells (cor = 0.388, *P* = 0.001078) and mast cells resting (cor = 0.386, *P* = 0.00108) while negatively correlated with resting NK cells (cor = −0.317, *P* = 0.007704) and CD4^+^ memory resting T cells (cor = −0.235, *P* = 0.04937) (**Figure 7C**). Moreover, ESTIMATE scores were compared between the OSG high- and OSG low-risk groups. The OSG high-risk group showed significantly lower immune scores, while stromal scores and total ESTIMATE scores did not differ significantly between the groups (**Figure 7D**).

### Association between risk score and clinical features

To further accurately assess the prognostic value of OSGs in clinical information, the tumor stage, patient age, gender, and race were used to analyze the correlation with overall survival in this study. Our results demonstrate that the high-risk group consistently exhibited a significantly higher risk score (**Supplementary Figure 1A**), regardless of whether patients were younger or older than 60 years. Although the overall survival between patients aged >60 and ≤60 did not show a statistically significant difference (**Supplementary Figure 1B**), further stratified analysis revealed that the high-risk group showed significantly poorer survival outcomes in patients ≤60 years (**Supplementary Figure 1C**). Similarly, in patients >60 years, the high-risk group also displayed a comparable and statistically significant association with adverse prognosis (**Supplementary Figure 1D**).

Similarly, in our sex-based subgroup analysis, we observed comparable findings. Although no statistically significant difference in prognosis was detected between male and female patients overall, further stratification revealed that high-risk status remained strongly associated with adverse outcomes in both groups (**Supplementary Figures 1E, F**). Among female patients, high-risk individuals similarly demonstrated higher risk scores and worse prognosis (**Supplementary Figure 1G**). Likewise, among male patients, the high-risk group exhibited significantly elevated risk scores and poorer survival outcomes (**Supplementary Figure 1H**).

Due to limitations in the clinical annotation of TCGA datasets, our race-stratified analysis was restricted to comparing “White” versus “Other” populations. Despite this broad categorization, consistent patterns emerged regarding high-risk patients who demonstrated significantly elevated risk scores and worse overall survival in the White subgroup (*P* = 0.000989, **Supplementary Figures 1I–K**).

Similarly, high-risk individuals of other racial subgroups showed poorer survival outcomes (*P* = 0.03089, **Supplementary Figure 1L**). Notably, our risk stratification model revealed consistent prognostic value in both subgroups. In early-stage (I–II) patients, high-risk individuals exhibited significantly elevated risk scores (mean score: 2.326 vs. 0.453; *P* = 1.1317E−06, **Supplementary Figure 1M**). When categorizing patients into early-stage (I–II) and late-stage (III–IV) disease, we observed that while the overall survival difference between these groups did not reach statistical significance, early-stage cases demonstrated a trend toward better prognosis (**Supplementary Figure 1N**, *P* = 0.2286). Additionally, early-stage patients within high-risk groups showed worse clinical outcomes (log-rank *P* = 0.000774, **Supplementary Figure 1O**). In advanced-stage (III–IV) patients, similar results showed comparable risk score differentiation (mean score: 3.123 vs. 0.543; *P* = 0.000105, **Supplementary Figure 1M**), with poorer survival in the high-risk group (log-rank *P* = 0.01491, **Supplementary Figure 1P**).

Additionally, we could calculate each patient’s total points and the corresponding overall survival probability using the constructed nomogram (**Supplementary Figure 1Q**). Calibration curves demonstrated high consistencies between the predicted overall survival and the actual overall survival rates at 1 year (**Supplementary Figure 1R**), 3 years (**Supplementary Figure 1S**), and 5 years (**Supplementary Figure 1T**). These results reinforced that our risk stratification model maintained robust predictive value across age, sex, race subgroups, and disease stages, potentially identifying high-risk patients who might benefit from more aggressive therapeutic interventions regardless of initial staging.

### CALM1 as a prognostic and diagnostic biomarker

A total of five key genes were screened using machine learning and prognosis analysis in the TCGA training dataset. Further prognostic analysis of individual genes in the GSE84976 (**Supplementary Figures 2A–E**) database demonstrated that there were four key genes with consistent correlation of overall survival, with genes having high expression showing poor overall survival, including ACACA (**Supplementary Figure 2A**, log-rank *P* = 0.000538), CALM1 (**Supplementary Figure 2B**, log-rank *P* = 0.019632), DNM2 (**Supplementary Figure 2C**, log-rank *P* = 0.03332), and POMC (**Supplementary Figure 2E**, log-rank *P* = 0.002665). HSP90B showed no significant correlation with either expression or overall survival in the GSE84976 validation data (**Supplementary Figure 2D**, log-rank *P* = 0.407925).

Moreover, the ICGC validation set also revealed consistent correlations between overall survival and three key genes: ACACA, CALM1, and DNM2, all showing high expression associated with poor prognosis (**Supplementary Figures 3A–C**), aligning with our initial results. HSP90B1 exhibited a discordant prognostic trend compared to our training data and GSE84976 validation cohort, with higher expression correlating with better survival in the ICGC data (*P* = 0.044791, **Supplementary Figure 3D**). POMC showed no significant correlation with either expression or overall survival in the ICGC validation cohort (*P* = 0.762312, **Supplementary Figure 3E**). Based on their reproducible prognostic associations, ACACA, CALM1, and DNM2 were prioritized for subsequent mechanistic studies.

The expression levels of key genes were determined in the TCGA training and ICGC testing data (**Supplementary Figures 4A–J**). The results revealed consistent expression between the high- and low-risk groups, including ACACA (**Supplementary Figures 4A,F**), CALM1 (**Supplementary Figures 4B, G**), DNM2 (**Supplementary Figures 4C, H**), and POMC (**Supplementary Figures 4E, J**). In our ICGC validation analyses, we observed a discordant expression pattern of HSP90B1 between the TCGA training set and the ICGC validation set. Specifically, HSP90B1 was significantly upregulated in the high-risk group within the TCGA cohort (*P* = 0.000159, **Supplementary Figure 4D**), whereas it showed higher expression in the low-risk group in the ICGC dataset (*P* = 3.611E−31, **Supplementary Figure 4I**). We opted not to include HSP90B1 in further analyses to ensure the robustness of our model.

In addition, ROC analysis was performed to evaluate diagnostic efficacy. In the TCGA training cohort, CALM1 demonstrated the highest AUC (0.9225), followed by POMC (AUC = 0.8556) and ACACA (AUC = 0.8356) (**Supplementary Figure 5A**). Similarly, in the ICGC validation data, CALM1 maintained high diagnostic performance (AUC = 0.8378) (**Supplementary Figure 5B**), exceeding the predefined threshold of AUC >0.8. We further evaluated the diagnosis of these genes using clinical samples, including three UVM tumor samples and matched adjacent normal tissues. The results demonstrated that CALM1 and HSP90B1 exhibited superior discriminatory power, with significantly higher AUC values compared to other candidate genes (**Supplementary Figure 5C**). Based on these findings, CALM1 was selected for further functional analysis to elucidate its biological role.

### The effect of H_2_O_2_ on the viability of UVM cells

**Figure 8:**
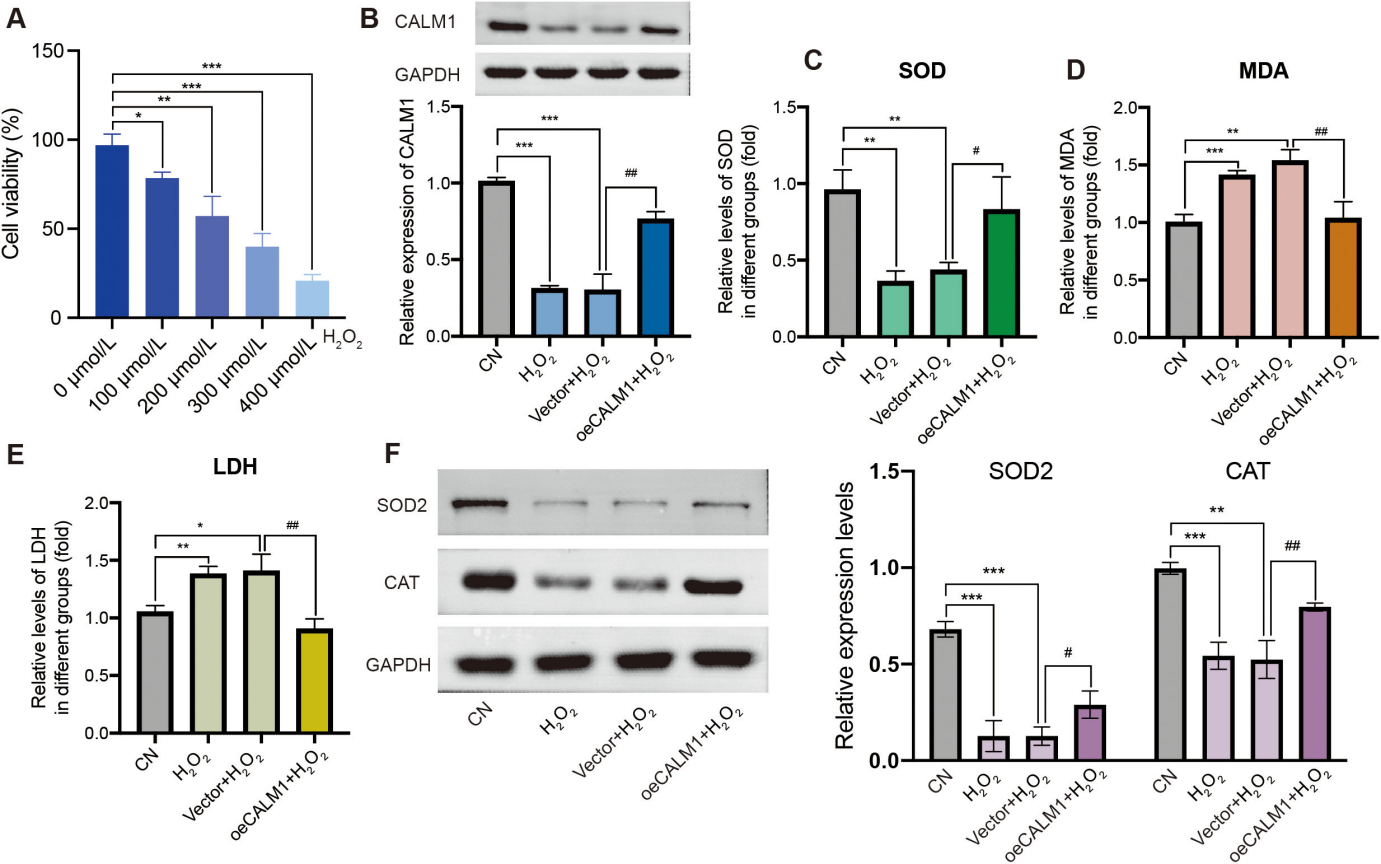
CALM1 overexpression attenuated H_2_O_2_-induced oxidative stress in MP65 cells. **(A)** Cell viability was inhibited by H_2_O_2_. **(B)** The expression level of CALM1 was detected by Western blot upon transfection of H_2_O_2_-induced MP65 cells with negative control (vector) or CALM1 overexpression. The activity of SOD **(C)**, MDA **(D)**, and LDH **(E)** in H_2_O_2_-induced MP65 cells was determined by ELISA. **(F)** Western blot evaluating the expression levels of SOD2 and CAT. * or ^#^ represents *P* < 0.05, ** or ^##^ represents *P* < 0.01, and *** or ^###^ represents *P* < 0.001. “0 µmol/L” denotes the untreated control.

To evaluate the impact of oxidative stress on cell viability, UVM cells (MP65, MM28) were treated with various concentrations of H_2_O_2_ (0, 100, 200, 300, and 400 µmol/L) for 24 h. Cell viability was assessed using the CCK-8 assay. The results indicated that cell viability decreased significantly in a dose-dependent manner in MP65 (**Figure 8A**) and MM28 (**Supplementary Figure 6A**). A concentration of 200 µmol/L (cell viability: 57.2% in MP65 and 47.3% in MM28) was selected for subsequent experiments.

### CALM1 overexpression attenuates H_2_O_2_-induced oxidative stress in UVM cells

To further investigate the role of CALM1 in H_2_O_2_-induced oxidative stress, UVM cells were transfected with the CALM1 overexpression vector (oeCALM1 group) or a control vector and then treated with H_2_O_2_. Western blot analysis confirmed low expression of CALM1 in H_2_O_2_-treated cells compared to the control (CN) group in MP65 cells (**Figure 8B**). In the H_2_O_2_-challenged environment (200 µmol/L, 24 h duration), the CALM1 overexpression system achieved a robust 1.44 ± 0.012-fold increase in CALM1 expression in MM28 (Western blot quantification, normalized to GAPDH, *P* = 5.36E−05, **Supplementary Figure 6B**), meeting the predetermined threshold for successful genetic manipulation. This overexpression efficiency remained stable throughout the oxidative stress exposure period (24 h post-treatment), establishing an appropriate experimental platform for probing CALM1’s functional role in redox regulation.

To evaluate the effect of CALM1 on oxidative stress, the levels of SOD, MDA, and LDH were measured using ELISA assays. H_2_O_2_ treatment significantly decreased SOD levels (*P* = 0.03698; **Figure 8C**) and increased MDA and LDH levels (*P* = 0.008023 and *P* = 0.007522, respectively; **Figures 8D, E**), compared to untreated controls (CN). Notably, oeCALM1 effectively reversed the reduction in SOD and suppressed the accumulation of MDA and LDH (**Figures 8C–E**) in H_2_O_2_-treated MP65 cells. Compared with the H_2_O_2_ negative control group (vector + H_2_O_2_), oeCALM1 also restored the expression of SOD2 (*P* = 0.02858) and CAT (*P* = 0.00918), both of which were suppressed by H_2_O_2_ treatment (**Figure 8F**). This protective pattern was consistently replicated in MM28 cells, where oeCALM1 similarly mitigated H_2_O_2_-mediated SOD suppression (50.39% recovery, *P* = 0.00046, **Supplementary Figure 6C**) and reduced oxidative damage markers (MDA: 23.53% decrease; LDH: 29.81% decrease; both *P* < 0.05 vs. H_2_O_2_-treated vector controls, **Supplementary Figures 6D, E**). Western blot results also revealed that oeCALM1 restored the expression of SOD2 (*P* = 0.02858) and CAT (*P* = 0.00918) in H_2_O_2_ treatment (**Supplementary Figure 6F**). The concordant results across both cell lines demonstrated the robust capacity of CALM1 to counteract oxidative stress regardless of cellular context.

### CALM1 overexpression attenuates H_2_O_2_-induced apoptosis in UVM cells

**Figure 9:**
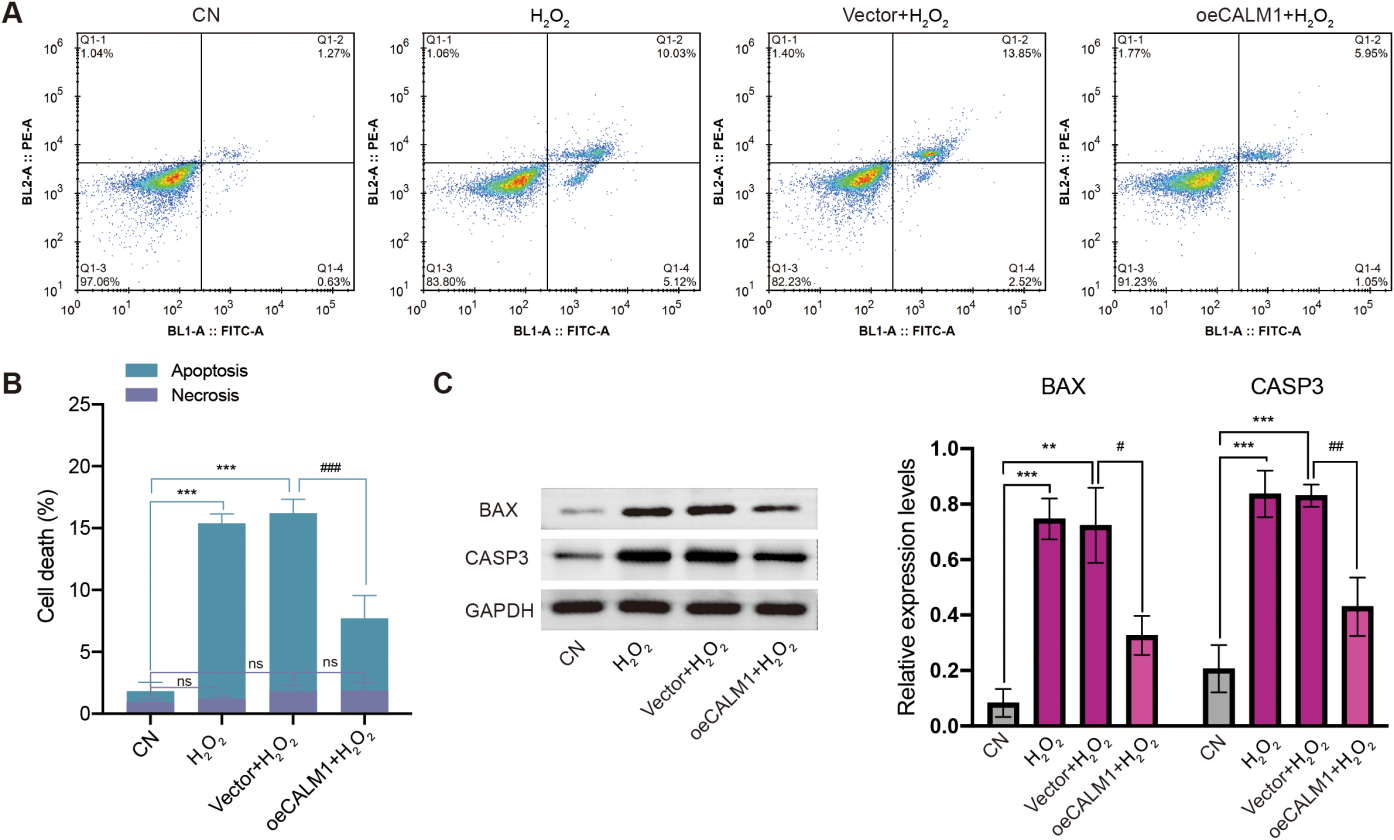
CALM1 overexpression attenuated H_2_O_2_-induced apoptosis in MP65 cells. **(A)** After transfection with negative control (vector) or CALM1 overexpression, MP65 cells were determined by Annexin V-FITC/propidium iodide (PI) staining. **(B)** Percentage of apoptotic cell death and necrosis. **(C)** The expression level of BAX and CASP3 (caspase 3) was detected by Western blot. Three independent experiments were carried out. “ns” represents no statistical significance; * or ^#^ represents *P* < 0.05, ** or ^##^ represents *P* < 0.01, and *** or ^###^ represents *P* < 0.001.

Next, we examined the effect of CALM1 on apoptosis induced by oxidative stress. Flow cytometry analysis showed that H_2_O_2_ significantly increased the rate of apoptosis in MP65 cells, compared to untreated controls (**Figure 9A**). Importantly, this effect was attenuated by oeCALM1 transfection (**Figures 9A, B**), while necrosis remained unaffected. Compared with the CN group, the expression levels of CASP3 (caspase-3; *P* = 0.000797) and BAX (*P* = 0.00021) were significantly elevated following H_2_O_2_ treatment (**Figure 9C**). Overexpression of CALM1 mitigated the H_2_O_2_-induced increases in CASP3 (*P* = 0.00355) and BAX (*P* = 0.01071) expression levels (**Figure 9C**). These protective effects were consistently observed in MM28 cells, where oeCALM1 similarly attenuated both the percentage of apoptotic cells (49.14% reduction, *P* = 0.000464, **Supplementary Figures 7A, B**) and the expression levels of CASP3 (50.60% decrease) and BAX (26.62% decrease) following H_2_O_2_ exposure (**Supplementary Figure 7C**).

### CALM1 overexpression attenuates H_2_O_2_-induced proliferation, migration, and invasion in UVM cells

Our investigation extended to examine the impact of CALM1 overexpression on proliferation, migration, and invasion under H_2_O_2_-induced oxidative stress (200 µmol/L, 24 h). CCK-8 assays demonstrated that oeCALM1 significantly enhanced cell proliferation by 37.58% (*P* = 0.00383, **Supplementary Figure 8A**) in MP65 cells and 43.35% (*P* = 0.00734, **Supplementary Figure 8B**) in MM28 cells at 72 h, effectively reversing the H_2_O_2_-mediated growth inhibition. This pro-proliferative effect was consistently observed across both cell lines. Furthermore, transwell assays revealed that oeCALM1 restored migratory capacity by 1.51-fold (*P* = 0.00288) in MP65 cells and 1.62-fold (*P* = 0.00436) in MM28 cells, compared to H_2_O_2_-treated controls (**Supplementary Figures 8C, D**). The oeCALM1 increased invasive potential by 1.84-fold (*P* = 0.00069) in MP65 cells and 2.22-fold (*P* = 6.013E−06) in MM28 cells, relative to oxidative stress conditions (**Supplementary Figures 8E, F**). The concordant results in both MP65 and MM28 cell lines established CALM1 as a multifunctional regulator capable of counteracting oxidative stress-induced impairment of critical oncogenic processes. These findings suggest CALM1 may serve as a key mediator in maintaining cellular functionality during redox imbalance.

## Discussion

UVM is a highly aggressive and potentially devastating form of ocular cancer, characterized by a poor prognosis and high metastatic potential (2). Despite advancements in early diagnosis and local treatment, the survival rate for patients with UVM remains dismally low, underscoring the urgent need for reliable biomarkers to enhance diagnostic accuracy and predict clinical outcomes (24–26). Recent research has increasingly focused on the role of oxidative stress in UVM pathogenesis, revealing that oxidative stress not only contributes to tumor progression but also impacts therapeutic resistance (19, 27, 28). Studies have identified key molecular pathways—such as the HIF-1 signaling axis—involved in regulating oxidative stress responses across various diseases (29, 30). Furthermore, elevated levels of ROS and disrupted antioxidant mechanisms have been observed in UVM, suggesting the presence of potential therapeutic targets (31, 32). However, the specific genes that respond to oxidative stress and influence UVM progression remain poorly characterized. The molecular mechanisms and key regulatory genes involved in this process require further elucidation. Therefore, deeper investigation into the interplay between oxidative stress and UVM biology could pave the way for novel diagnostic tools and targeted therapies to improve patient outcomes.

In this study, we identified 185 oxidative stress-related DEGs between control and UVM samples. Among them, 15 intersecting genes were identified between hub genes from the PPI networks and prognostic genes. Multiple machine learning algorithms (LASSO regression, random forest, SVM, NNET, GMB, and XGBoost) were employed to identify novel diagnostic genes, including ACACA, CALM1, HSP90B1, DNM2, and POMC. These five genes were subsequently used to construct a prognostic risk signature model. Patients classified as high risk exhibited poorer prognoses and demonstrated elevated immune infiltration scores for CD4^+^ memory-activated T cells, activated NK cells, and resting mast cells. Additionally, functional experiments showed that CALM1 overexpression attenuated H_2_O_2_-induced oxidative stress and apoptosis in MP65 cells.

ACACA (acetyl-CoA carboxylase alpha) is a biotin-containing enzyme that catalyzes the carboxylation of acetyl-CoA to malonyl-CoA (33), participating in lipid metabolism and acetyl-CoA metabolic processes (34). Downregulation of ACACA suppresses the malignant progression of prostate cancer (35). In hepatocellular carcinoma, ACACA has been identified as a central gene associated with poor prognosis (36). Consistent with these findings, our results indicated that high ACACA expression was associated with worse prognosis in UVM patients. Interestingly, its downregulation in lung fibroblasts was found to trigger an inflammatory phenotypic shift (37). CALM1 (calmodulin 1) is a member of the EF-hand calcium-binding protein family, involved in G2/M cell cycle transition and calcium signal transduction by modulating a wide range of enzymes (38–40). CALM1 has shown high diagnostic and prognostic value in several cancers, including UVM, bladder cancer, and breast cancer (41), which aligns with our findings linking high CALM1 expression to poor overall survival in UVM patients. Prior studies reported a positive correlation between CALM1 expression and macrophage/neutrophil infiltration in skin cutaneous melanoma (41). Our study supports this, suggesting CALM1 may modulate the tumor microenvironment. Furthermore, in esophageal squamous cell carcinoma, CALM1 was shown to promote tumor progression and reduce sensitivity to EGFR inhibitors (42). In ovarian cancer, it was associated with tumor classification and immune status (43). These findings are consistent with our observation that CALM1 overexpression alleviated H_2_O_2_-induced oxidative stress, reduced MDA and LDH accumulation, and inhibited apoptosis in UVM cells. CALM1 may regulate tumor apoptosis through multiple signaling pathways. It activates the Ca^2+^/calmodulin-dependent protein kinase II (CaMKII) pathway, inhibits mitochondrial apoptosis by reducing the Bax/Bcl-2 ratio, and promotes glioma cell survival (44). CALM1 can also activate the NF-κB pathway by binding to IκB kinase (IKK) (45), upregulating anti-apoptotic proteins to inhibit prostate cancer apoptosis (46). Further studies have shown that activated Ca^2+^/CaMKII can enhance CaMKII/NF-κB interaction and NF-κB activation (47). In addition, CALM1 has been implicated in AKT regulation, especially in PIK3CA-mutated breast cancer (48). The activation of CaMKII also promotes PI3K/Akt signaling, facilitating anti-apoptotic mechanisms in prostate cancer (49). This pathway enhances glucose uptake, promotes glycolysis, and inhibits apoptosis in hepatocellular carcinoma (50). Although CALM1 is known to inhibit apoptosis in many cancers, its role in UVM apoptosis remains underexplored. Notably, CALM1 may activate Ca^2+^/CaMKII signaling, while excessive Ca^2+^ can induce apoptosis in some cancer types (51). In GNAQ/11-mutant UVM cells, mutations increase cytosolic calcium and trigger p53-dependent apoptosis (52). Moreover, reduced Ca^2+^ flux in BAP1^+/−^ cells hinders apoptosis despite DNA damage accumulation in UVM with germline BAP1-inactivating mutations (53).

CALM1 is involved in the process of oxidative stress through the Ca^2+^ signaling pathway. Oxidative stress could activate the TRPM8 channel to induce Ca^2+^ and pro-apoptotic signals in prostate cancer (54). Oxidative stress activated the Ca^2+^-CaMKII cascade to inhibit early autophagy induction, which led to mitochondria fragmentation and loss of mitochondrial membrane potential (55). Mitochondrial calcium uniporter (MCU)-mediated oxidative stress could increase mitochondrial calcium and decrease mitochondrial membrane potential in osteoblasts (56). The activation of the iNOS pathway led to higher reactive oxygen species and nitric oxide production, which accelerated gastric cancer cell apoptosis (57). The activation of oxidative stress induced the proliferation of leukemia cancer cells through cytosolic Ca^2+^ influx (58). The dysregulation of cytosolic Ca^2+^ also decreased mitochondrial function and increased oxidative stress (59). The mitochondrial oxidative stress was induced by *Clostridium botulinum* neurotoxin A via activation of the Ca^2+^ signaling pathway in neuroblastoma and glioblastoma tumor (60). The activated Ca^2+^/CAMKII axis increased NOX4 expression, creating a feedforward loop of oxidative damage (60). Additionally, Ca^2+^ signaling mediated airway inflammation in response to oxidative stress through activation of the ERK pathway (61). These findings revealed that Ca^2+^ signaling served as a central regulator of oxidative stress responses. HSP90B1 (heat shock protein 90 beta family member 1) encodes an ATP-dependent molecular chaperone involved in protein stabilization and folding (62). Previous studies have suggested that HSP90B1 is a promising candidate for cancer diagnosis and prognosis (63) and plays a role in regulating cisplatin sensitivity in bladder cancer (64). DNM2 (dynamin 2) is a member of the GTPase protein family (65). It has been associated with poor prognosis in various cancers (66). Interestingly, downregulation of DNM2 was linked to worse outcomes and older age in neuroblastoma patients (67). Our findings are consistent, showing that higher DNM2 expression correlates with worse survival in UVM. POMC (proopiomelanocortin) is involved in physiological processes including pigmentation and inflammation (68). It has been identified as a survival-related gene in colorectal cancer (69). Moreover, POMC-negative expression was associated with better response to paclitaxel and carboplatin chemotherapy in lung cancer (70).

Nowadays, the diversification of treatment strategies has contributed to improved clinical outcomes for patients. Clinical trial results have demonstrated that anti-PD-1 antibodies can achieve durable clinical benefits in patients with UVM (71). Oxidative stress has been reported to regulate programmed death-ligand 1 (PD-L1) expression on tumors, thereby influencing tumor responses to immune checkpoint inhibitors (72). In our analysis, significant differences in immune cell infiltration levels were observed between the OSG high-risk and OSG low-risk groups. Elevated infiltration scores of CD4^+^ memory-activated T cells and activated NK cells were found in the high-risk group, suggesting that CD4^+^ T lymphocytes and NK cells may contribute to antitumor activity in UVM (73, 74). Dysregulated T-cell infiltration may alter antigen presentation to CD4^+^ T cells and impair epitope recognition, potentially contributing to autoimmune or inflammatory diseases (75). T-cell homeostasis has been associated with Fas-mediated apoptosis (76). Our results showed a negative correlation between CD4^+^ memory resting T cells and ACACA expression (*r* = −0.25, *P* = 0.042). Inhibition of ACACA function has been reported to enhance memory CD4^+^ T-cell formation via fatty acid metabolism (77), and ACACA has also been shown to suppress Th9 differentiation in naive CD4^+^ T cells through fatty acid synthesis (78). CALM1 was positively correlated with CD4^+^ T cells (*r* = 0.405, *P* = 0.001), which aligns with previous findings (79). Although CALM1 has not been directly studied in T lymphocytes, its known role in Ca^2+^ signaling suggests possible involvement in T-cell functions such as activation and differentiation (80), including CD8^+^ and CD4^+^ T cells (81). Memory and recall responses by CD8^+^ T cells require Ca^2+^ channel expression in CD4^+^ T cells (82). Furthermore, exosomal regulation of Ca^2+^ signaling has been shown to reduce CD40L expression and suppress CD4^+^ T-cell activation and proinflammatory cytokine secretion (83). Differentiation of naive CD4^+^ T cells into Th17 and Treg subsets is also dependent on Ca^2+^ signaling (84).

Other studies have indicated that CD8^+^ T cells, macrophages, and NK cell infiltration are associated with poor prognosis in UVM (74, 85). Hepatic NK cells have been shown to occupy the same niche as uveal melanoma micrometastases in the liver sinusoids (86). Low-risk primary UVM tumors are characterized by reduced HLA class I expression and increased NK cell infiltration, which is associated with a decreased risk of disease recurrence (87). Consistent with these findings, our results also revealed high levels of resting NK cell infiltration in low-risk UVM patients (**Figure 7A**). Calcium channels involved in Ca^2+^ signaling have been found to regulate the homeostasis of secretory lysosomes and their interaction with mitochondria in human NK cells (88). Deletion of calcium channels in NK cells has been shown to impede autophagic flux and lead to the accumulation of dysfunctional mitochondria, contributing to increased oxidative stress (88). Moreover, our results showed that immune scores were lower in the high-risk group, indicating poorer prognosis—contrary to findings in gastric cancer (89). This oxidative stress-related prognostic model may therefore have value in guiding clinical immunotherapy for UVM.

In our analysis, the dysregulated genes were significantly enriched in several critical signaling pathways, including apoptosis, FoxO, and HIF-1 pathways. These pathways are closely associated with oxidative stress in tumors and play crucial roles in tumorigenesis and progression. Prior studies have shown that FoxO transcription factor knockdown reduces UVM cell proliferation (90), while inhibition of FoxO3a promotes proliferation and invasion in UVM cells (91). Similarly, knockdown of HIF-1 significantly impairs UVM tumor progression (92), and silencing HIF-1α reduces UVM cell migration, invasion, and adhesion (93). Increased HIF-1α expression has also been associated with worse prognosis in UVM patients (94), suggesting that the FoxO and HIF-1 signaling pathways contribute to UVM progression. Notably, our findings highlight a particularly strong association between these pathways and UVM. The enrichment of dysregulated genes in the apoptosis, FoxO, and HIF-1 pathways underscores the critical role of oxidative stress regulation in UVM biology. Consistent with our results, other studies have shown that ROS production, oxidative stress signaling, and FoxO activity are pivotal in cancer development and progression (95). The uncontrolled activation of antioxidant signaling has been implicated in breast cancer progression via HIF-1 and FoxO pathways (96). Kim et al. demonstrated that FoxO3a acts as an anti-melanogenic factor mediating antioxidant-induced depigmentation, thereby influencing melanogenesis (97). Although no reports have directly linked the FoxO pathway to antioxidant activity in UVM, loss of FoxO function has been shown to confer growth and survival advantages to melanoma cells (98). Additionally, apoptosis under physiological conditions is tightly regulated by oxidative stress (99), and impaired signaling may disrupt the balance of apoptosis, contributing to tumor development (100). Our results revealed that the dysregulated genes were enriched in key pathways—FoxO, HIF-1, and PI3K-Akt—all of which are closely linked to oxidative stress regulation. Previous studies have reported that the PI3K signaling pathway is enriched in high-risk UVM groups and associated with worse prognosis (101). PI3K-Akt pathway-related risk scores have been shown to reflect distinct immune statuses and mutation landscapes in UVM patients (102). Furthermore, activation of the PI3K-Akt pathway and enhancement of DNA damage response mechanisms may help mitigate treatment side effects in UVM (103). Further investigation of these signaling pathways could provide deeper insights into the molecular mechanisms driving UVM progression and help identify novel therapeutic targets.

However, several limitations must be acknowledged in this study. First, although numerous novel oxidative stress-related biomarkers were identified as prognostic genes for predicting overall survival in UVM—and an external validation cohort was employed—these findings were derived entirely from bioinformatics analysis. Larger sample sizes are needed to further validate the robustness of these results. Second, potential biases may have been introduced by the machine learning algorithms, including overfitting and limitations inherent to database selection. Future research should aim to confirm the effectiveness of this five-biomarker-based diagnostic model using larger, independent cohorts. Third, the current database does not contain treatment-related information, making it impossible to incorporate this factor in the present study. We acknowledge this limitation and will ensure that treatment history is systematically recorded in future clinical sample collections to allow for more comprehensive analyses in subsequent research. Fourth, while we have successfully collected and analyzed RNA expression data from three UVM tissue samples and three paired adjacent normal tissues for preliminary validation, we acknowledge that the current sample size is insufficient to perform statistically meaningful survival prognosis analysis. The limited number of UVM cases (*n* = 3) lacks the statistical power required to correlate gene expression patterns with clinical outcomes such as overall survival. This preliminary analysis was primarily designed as a proof-of-concept validation of RNA expression trends observed in our larger genomic datasets.

## Conclusion

In conclusion, this study comprehensively investigated the role of OSGs in UVM, revealing their significant involvement in key signaling pathways—including HIF-1, FoxO, PI3K-Akt, and apoptosis—which are closely associated with tumor progression and oxidative stress regulation. Through differential gene expression analysis, protein–protein interaction networks, and multiple machine learning algorithms, we identified and validated several prognostic hub OSGs, including ACACA, CALM1, DNM2, POMC, and HSP90B1, which were all associated with poor survival outcomes in UVM patients. A robust risk signature model was constructed, demonstrating strong predictive accuracy for overall survival, particularly for 5-year prognosis. Furthermore, our findings emphasized the impact of risk scores on immune infiltration, with high-risk groups exhibiting distinct immune cell profiles. Experimental validation further confirmed the protective role of CALM1 in alleviating H_2_O_2_-induced oxidative stress and apoptosis in UVM cells. CALM1 upregulation also mitigated the inhibitory effects of H_2_O_2_ on key cellular processes, including proliferation, migration, and invasion, highlighting its potential as a therapeutic target. Collectively, these findings provide valuable insights into the molecular mechanisms underlying UVM and offer promising avenues for the development of targeted therapies and prognostic biomarkers.

## Data Availability

The original contributions presented in the study are included in the article/**Supplementary Material**. Further inquiries can be directed to the corresponding author.

## Supplementary material

Supplementary Figure 1 Combining the OSGs and clinical variables. **(A)** The difference of risk scores in patients with age > 60 and age≤ 60. **(B)** The survival curve showing no significant difference between age > 60 and age≤ 60. **(C)** The survival curve in age≤ 60 patients. **(D)** The survival curve in age> 60 patients. **(E)** The difference of risk scores in female and male patients. **(F)** The survival curve showing no significant difference between female and male. **(G)** The survival curve in female patients. **(H)** The survival curve in male patients. **(I)** The difference of risk scores in different race. **(J)** The survival curve showing no significant difference between white patients and others. **(K)** The survival curve in white patients. **(L)** The survival curve in non-white patients. **(M)** The difference of risk scores in different stages. **(N)** The survival curve showing no significant difference between early stage and advance stage patients. **(O)** The survival curve in early stage patients. **(P)** The survival curve in advance stage patients. **(Q)** Nomogram for predicting the 1-year, 3-years and 5-years overall survival. Calibration plot of the nomogram for predicting 1-year **(R)**, 3-years **(S)** and 5-years **(T)**.

Supplementary Figure 2 The correlation of five genes and overall survival in GSE84976 validation dataset. Survival curve showing the prognostic values of five genes of risk model, including ACACA **(A)**, CALM1 **(B)**, DNM2 **(C)**, HSP90B1 **(D)**, POMC **(E)**.

Supplementary Figure 3 The correlation of five genes and overall survival in ICGC validation dataset. Survival curve showing the prognostic values of five genes of risk model, including ACACA **(A)**, CALM1 **(B)**, DNM2 **(C)**, HSP90B1 **(D)**, POMC **(E)**.

Supplementary Figure 4 The relative expression levels of five OSGs in training and validation dataset. The genes expression in TCGA training dataset: ACACA **(A)**, CALM1 **(B)**, DNM2 **(C)**, HSP90B1 **(D)**, POMC **(E)**. The genes expression in ICGC testing dataset: ACACA **(F)**, CALM1 **(G)**, DNM2 **(H)**, HSP90B1 **(I)**, POMC **(J)**.

Supplementary Figure 5 ROC curves showing the comparisons. **(A)** ROC curves of ACACA, POMC, HSP90B1, CALM1, DNM2 in TCGA cohort as training cohort. **(B)** ROC curves of ACACA, POMC, HSP90B1, CALM1, DNM2 in ICGC cohort as testing dataset. **(C)** ROC curves of ACACA, CALM1, POMC, HSP90B, DNM2 in clinical samples as testing dataset.

Supplementary Figure 6 CALM1 overexpression attenuated H_2_O_2_-induced oxidative stress in MM28 cells. **(A)** Cell viability was inhibited by H_2_O_2_. **(B)** The expression level of CALM1 was detected by western blot upon transfection of H_2_O_2_-induced MM28 cells with negative control (vector) or CALM1 overexpression. The activity of SOD **(C)**, MDA **(D)** and LDH **(E)** in H_2_O_2_-induced MM28 cells was determined by ELISA. **(F)** Western blot evaluating the expression levels of SOD2 and CAT using western blot. * or ^#^ represents P<0.05, ** or ^##^ represents P<0.01, *** or ^###^ represents P<0.001. “0 µmol/L” denotes the untreated control.

Supplementary Figure 7 CALM1 overexpression attenuated H_2_O_2_-induced apoptosis in MM28 cells. **(A)** After transfection with negative control (vector) or CALM1 overexpression, MM28 cells were determined by Annexin V-FITC/propidium iodide (PI) staining. **(B)** Percentage of apoptotic cell death and necrosis. **(C)** The expression level of BAX and CASP3 (caspase 3) were detected by western blot. Three independent experiments were carried out. “ns” represents no statistical significance; * or ^#^ represents P<0.05, ** or ^##^ represents P<0.01, *** or ^###^ represents P<0.001.

Supplementary Figure 8 Effects of CALM1 on the proliferation, migration and invasion in UVM cells. **(A)** The proliferation curve of MP65 cells in different group. **(B)** The proliferation curve of MM28 cells in different group. **(C)** The micrographs of migration assay in MP65 and MM28 cells. **(D)** box plot representation of the migration assay analysis in MP65 and MM28 cells. **(E)** Images of invasion assay in MP65 and MM28 cells. **(F)** box plot of the invasion assay in MP65 and MM28 cells.
