# Supplementary figures and images for: Oxidative stress-related genes in uveal melanoma: the role of CALM1 in modulating oxidative stress and apoptosis and its prognostic significance
Source: Front Oncol. 2025 Aug 1;15:1618601. doi: 10.3389/fonc.2025.1618601 (PMC12353749; doi:10.3389/fonc.2025.1618601)

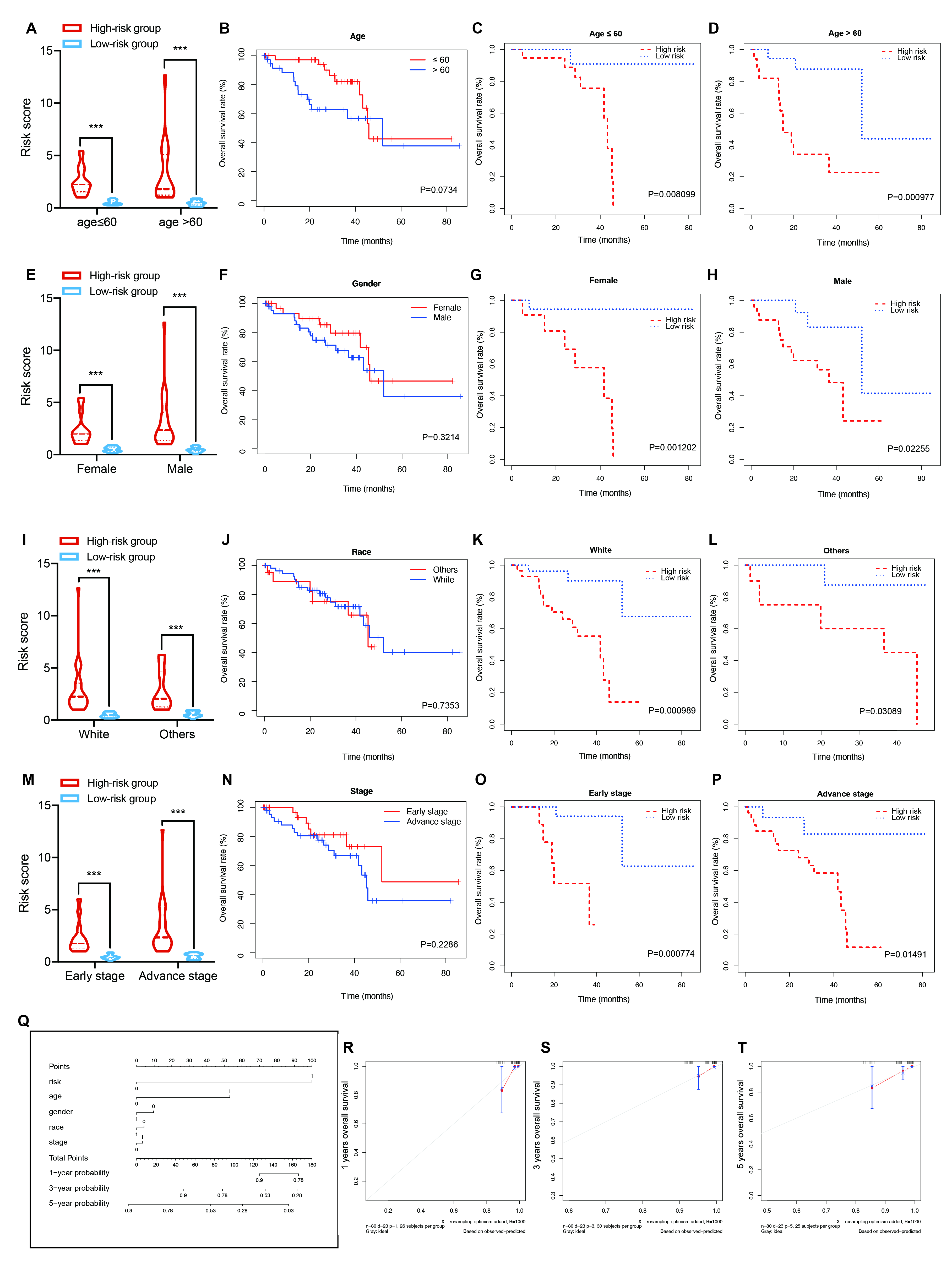

Supplement: Supplementary Figure 1 — Combining the OSGs and clinical variables. (A) The difference of risk scores in patients with age > 60 and age≤ 60. (B) The survival curve showing no significant difference between age > 60 and age≤ 60. (C) The survival curve in age≤ 60 patients. (D) The survival curve in age> 60 patients. (E) The difference of risk scores in female and male patients. (F) The survival curve showing no significant difference between female and male. (G) The survival curve in female patients. (H) The survival curve in male patients. (I) The difference of risk scores in different race. (J) The survival curve showing no significant difference between white patients and others. (K) The survival curve in white patients. (L) The survival curve in non-white patients. (M) The difference of risk scores in different stages. (N) The survival curve showing no significant difference between early stage and advance stage patients. (O) The survival curve in early stage patients. (P) The survival curve in advance stage patients. (Q) Nomogram for predicting the 1-year, 3-years and 5-years overall survival. Calibration plot of the nomogram for predicting 1-year (R), 3-years (S) and 5-years (T). [file Image1.tif]

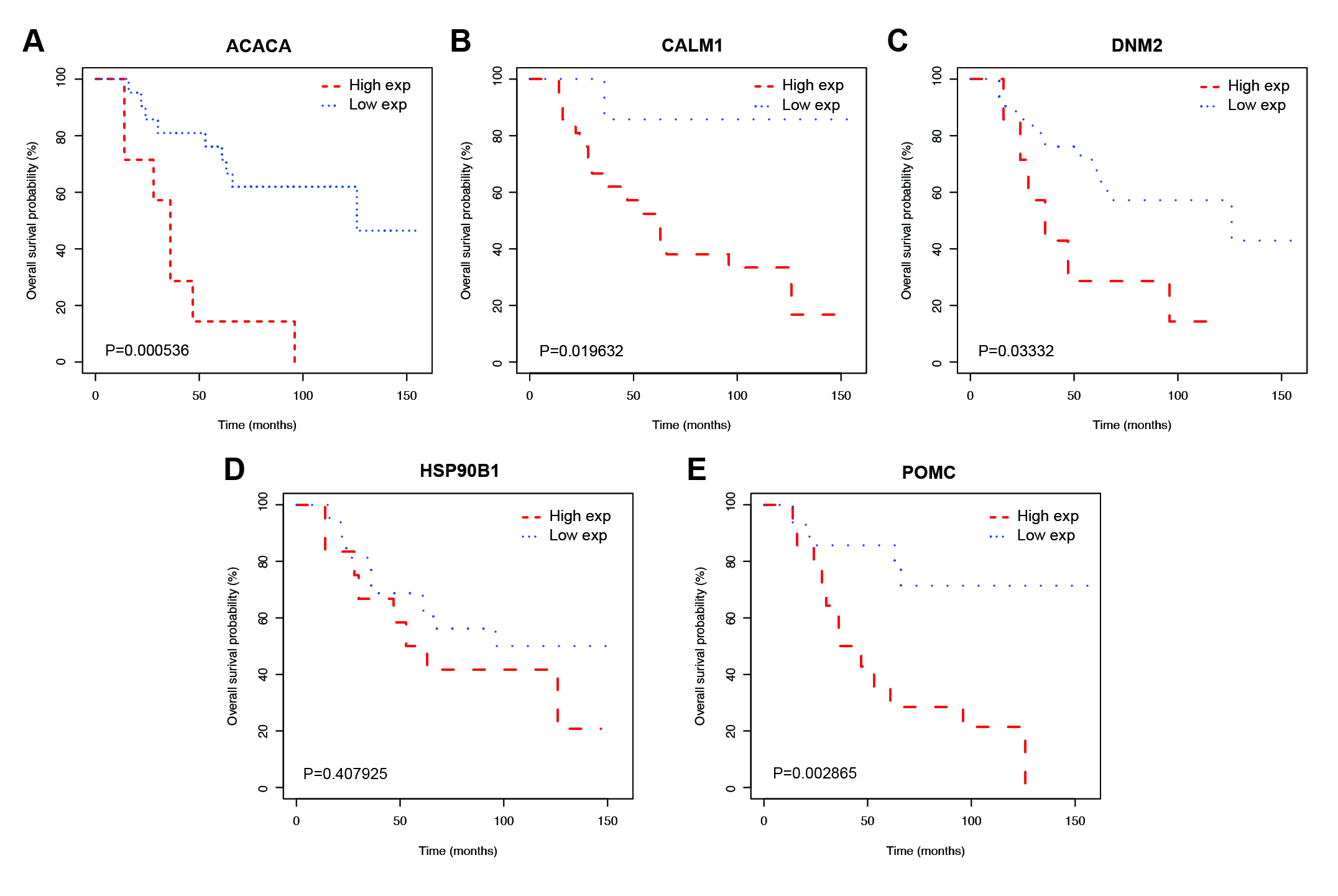

Supplement: Supplementary Figure 2 — The correlation of five genes and overall survival in GSE84976 validation dataset. Survival curve showing the prognostic values of five genes of risk model, including ACACA (A), CALM1 (B), DNM2 (C), HSP90B1 (D), POMC (E). [file Image2.tif]

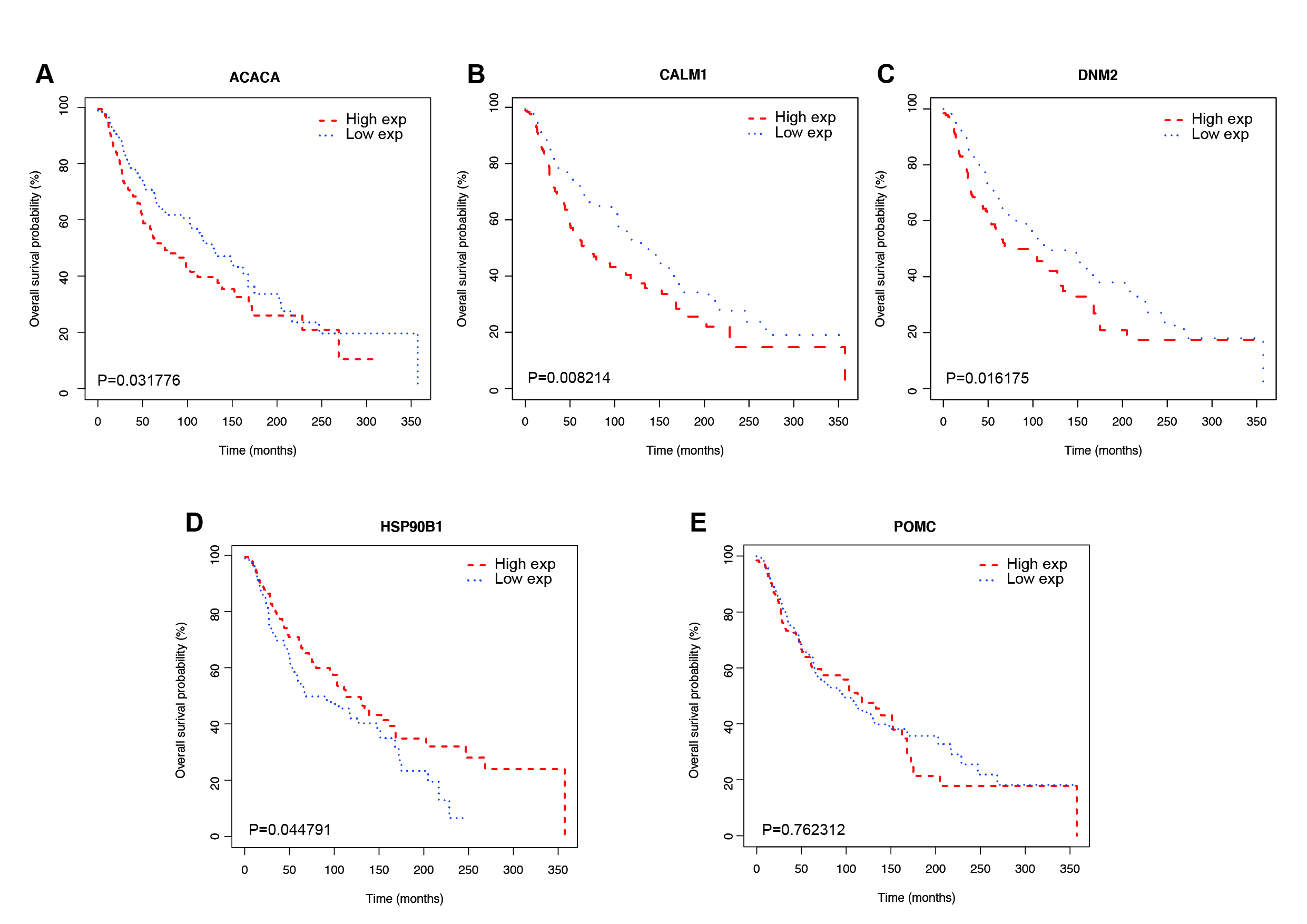

Supplement: Supplementary Figure 3 — The correlation of five genes and overall survival in ICGC validation dataset. Survival curve showing the prognostic values of five genes of risk model, including ACACA (A), CALM1 (B), DNM2 (C), HSP90B1 (D), POMC (E). [file Image3.tif]

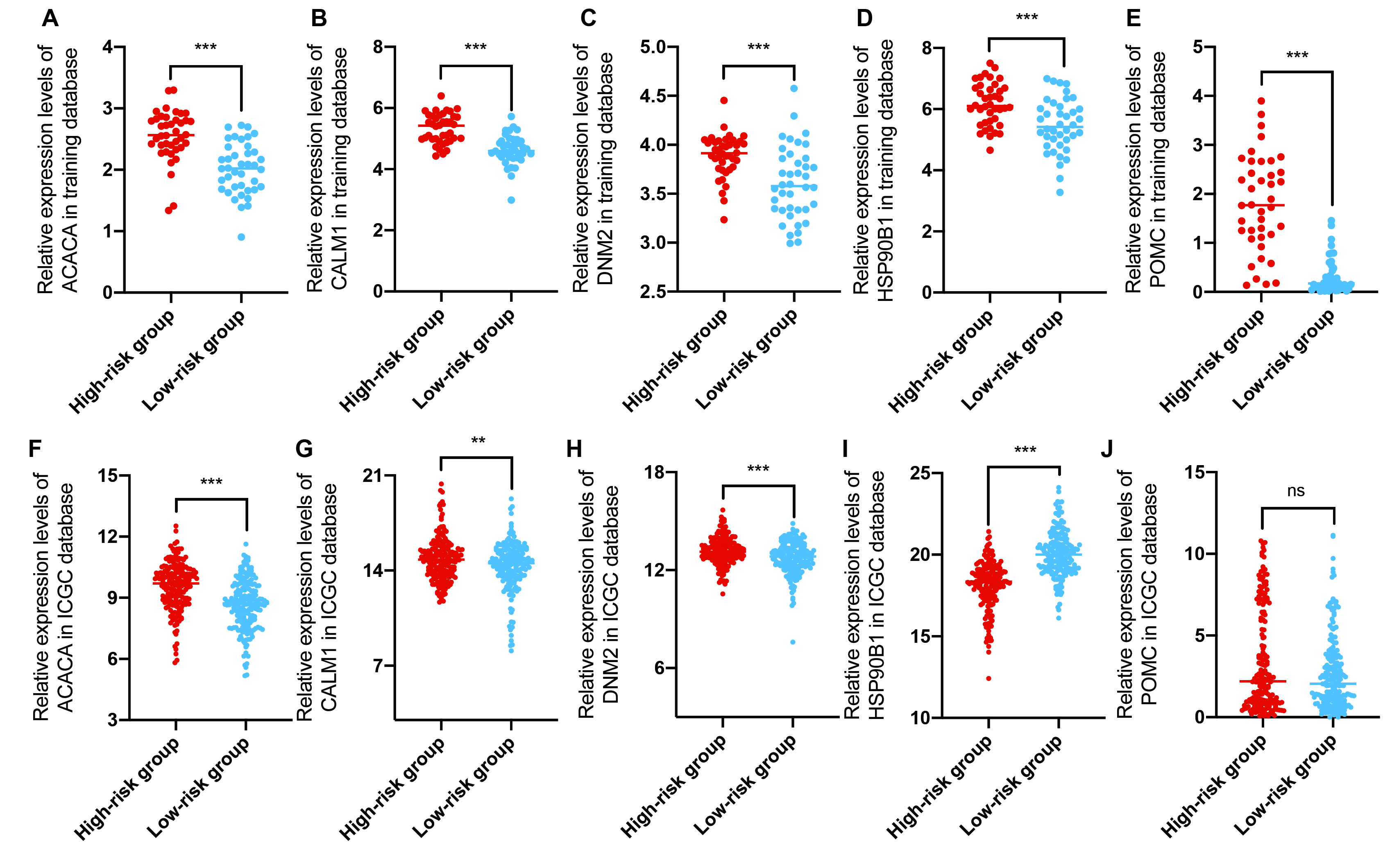

Supplement: Supplementary Figure 4 — The relative expression levels of five OSGs in training and validation dataset. The genes expression in TCGA training dataset: ACACA (A), CALM1 (B), DNM2 (C), HSP90B1 (D), POMC (E). The genes expression in ICGC testing dataset: ACACA (F), CALM1 (G), DNM2 (H), HSP90B1 (I), POMC (J). [file Image4.tif]

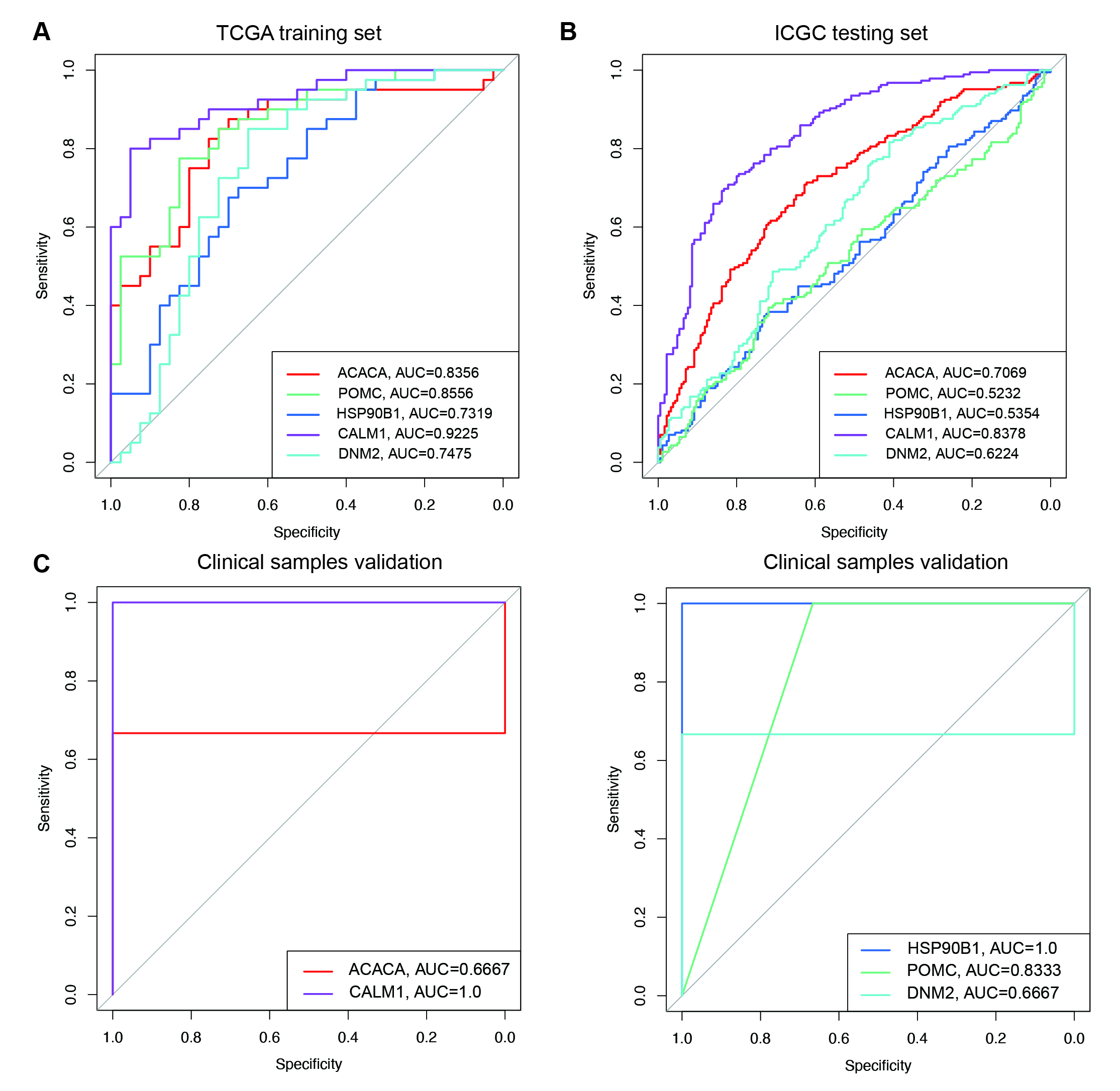

Supplement: Supplementary Figure 5 — ROC curves showing the comparisons. (A) ROC curves of ACACA, POMC, HSP90B1, CALM1, DNM2 in TCGA cohort as training cohort. (B) ROC curves of ACACA, POMC, HSP90B1, CALM1, DNM2 in ICGC cohort as testing dataset. (C) ROC curves of ACACA, CALM1, POMC, HSP90B, DNM2 in clinical samples as testing dataset. [file Image5.tif]

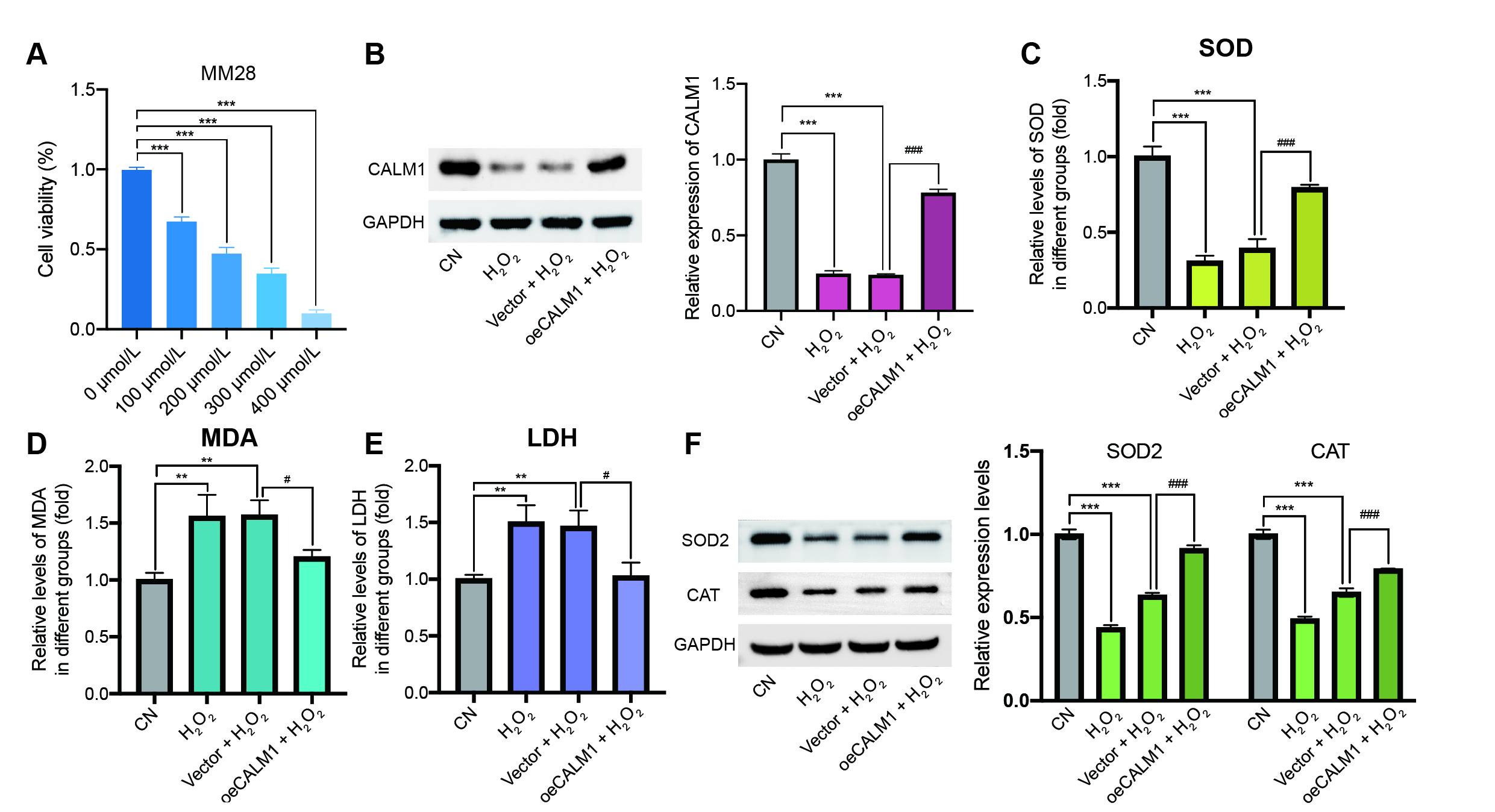

Supplement: Supplementary Figure 6 — CALM1 overexpression attenuated H2O2-induced oxidative stress in MM28 cells. (A) Cell viability was inhibited by H2O2. (B) The expression level of CALM1 was detected by western blot upon transfection of H2O2-induced MM28 cells with negative control (vector) or CALM1 overexpression. The activity of SOD (C), MDA (D) and LDH (E) in H2O2-induced MM28 cells was determined by ELISA. (F) Western blot evaluating the expression levels of SOD2 and CAT using western blot. * or # represents P<0.05, ** or ## represents P<0.01, *** or ### represents P<0.001. “0 µmol/L” denotes the untreated control. [file Image6.tif]

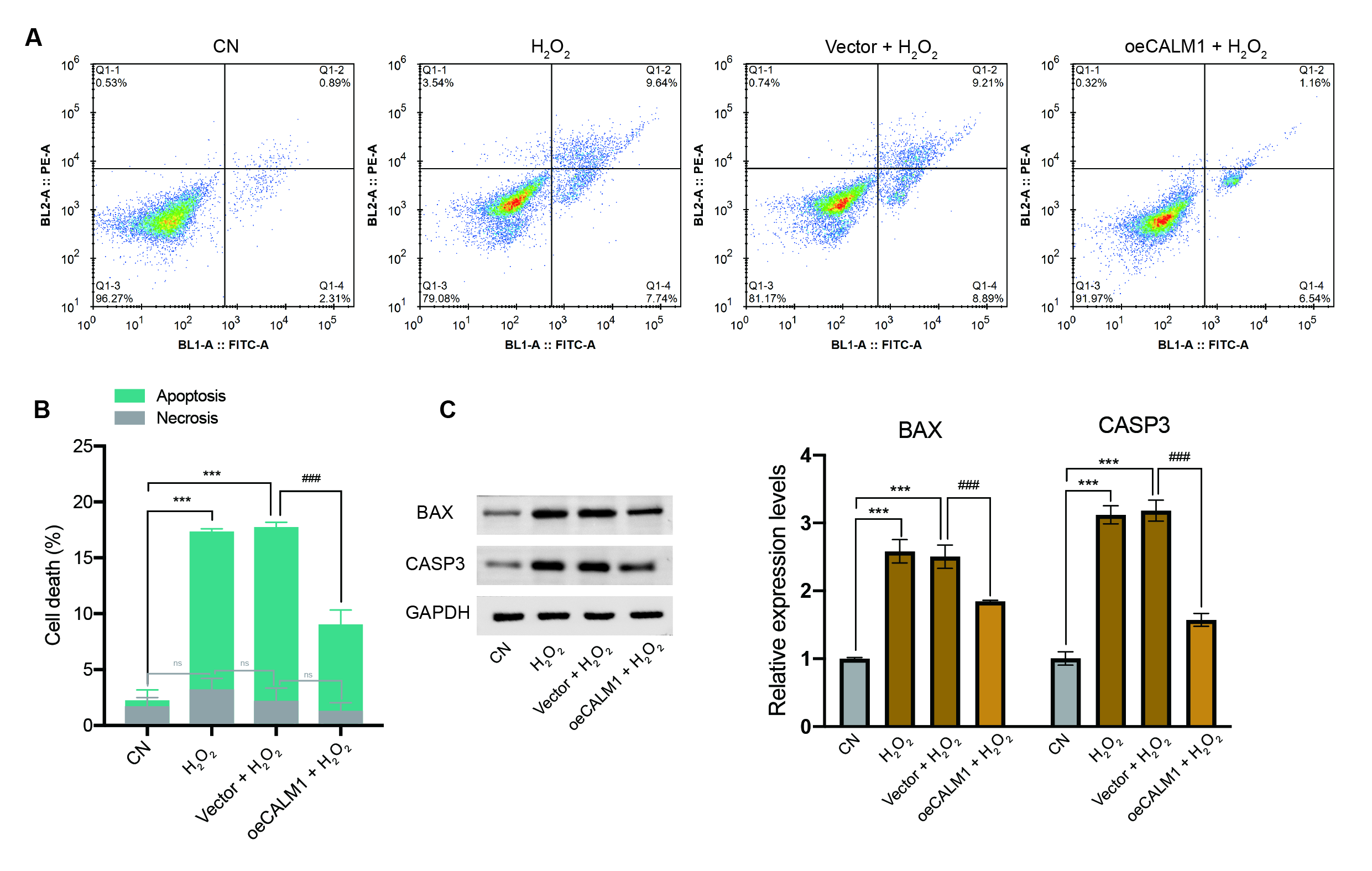

Supplement: Supplementary Figure 7 — CALM1 overexpression attenuated H2O2-induced apoptosis in MM28 cells. (A) After transfection with negative control (vector) or CALM1 overexpression, MM28 cells were determined by Annexin V-FITC/propidium iodide (PI) staining. (B) Percentage of apoptotic cell death and necrosis. (C) The expression level of BAX and CASP3 (caspase 3) were detected by western blot. Three independent experiments were carried out. “ns” represents no statistical significance; * or # represents P<0.05, ** or ## represents P<0.01, *** or ### represents P<0.001. [file Image7.tif]

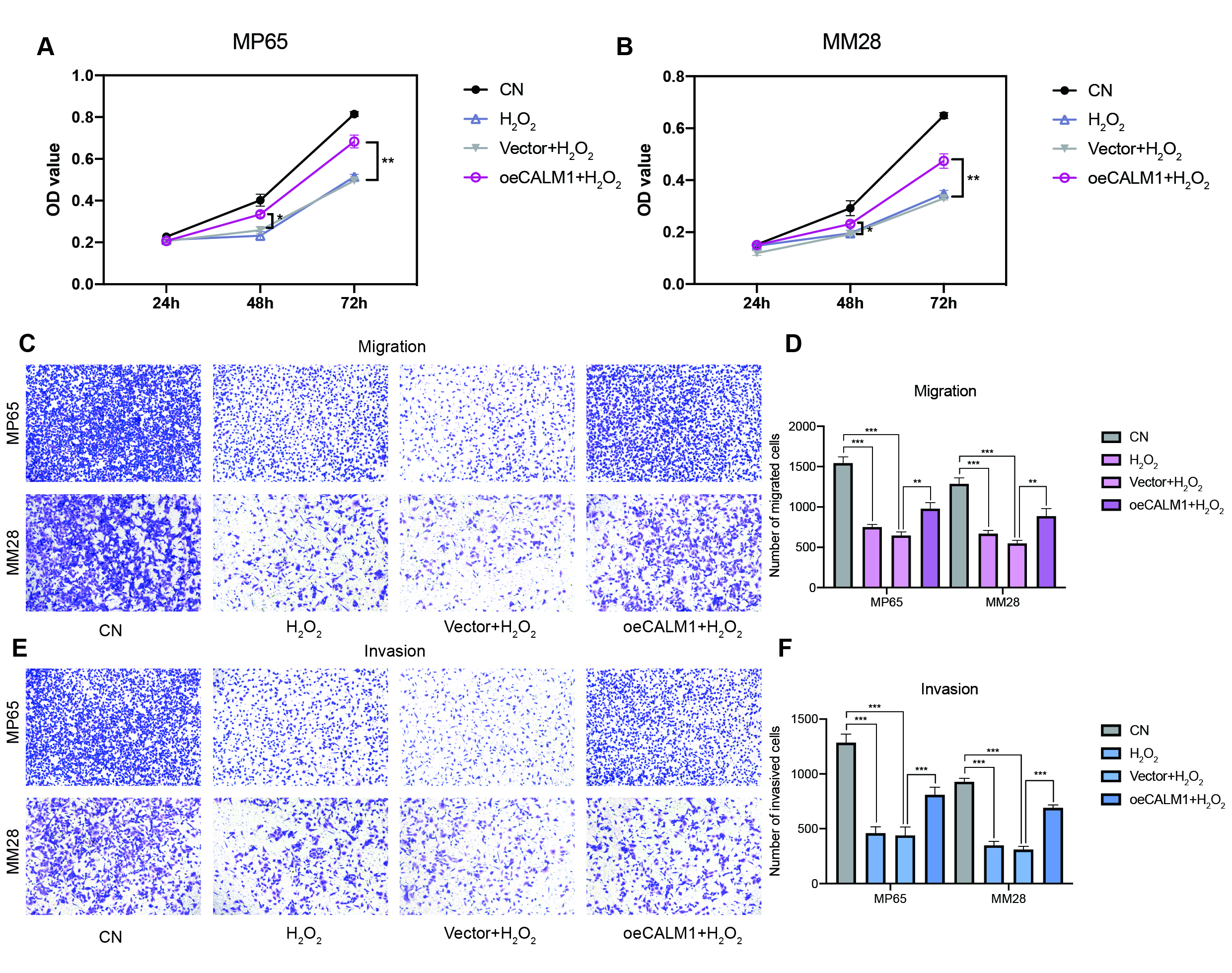

Supplement: Supplementary Figure 8 — Effects of CALM1 on the proliferation, migration and invasion in UVM cells. (A) The proliferation curve of MP65 cells in different group. (B) The proliferation curve of MM28 cells in different group. (C) The micrographs of migration assay in MP65 and MM28 cells. (D) box plot representation of the migration assay analysis in MP65 and MM28 cells. (E) Images of invasion assay in MP65 and MM28 cells. (F) box plot of the invasion assay in MP65 and MM28 cells. [file Image8.tif]
